# Cell-free synthesis of functional antibodies using a coupled *in vitro* transcription-translation system based on CHO cell lysates

**DOI:** 10.1038/s41598-017-12364-w

**Published:** 2017-09-20

**Authors:** M. Stech, O. Nikolaeva, L. Thoring, W. F. M. Stöcklein, D. A. Wüstenhagen, M. Hust, S. Dübel, S. Kubick

**Affiliations:** 1 Fraunhofer Institute for Cell Therapy and Immunology (IZI), Branch Bioanalytics and Bioprocesses (IZI-BB), Am Mühlenberg 13, 14476 Potsdam, Germany; 2 Technische Universität Berlin, Institut für Biotechnologie, Medizinische Biotechnologie, Gustav-Meyer-Allee 25, 13355 Berlin, Germany; 3 Technische Universität Braunschweig, Institute for Biochemistry, Biotechnology and Bioinformatics, Department of Biotechnology, Spielmannstr. 7, 38106 Braunschweig, Germany

## Abstract

Antibodies are indispensable tools for basic research as well as diagnostic and therapeutic applications. Consequently, the development of alternative manufacturing strategies which circumvent the hurdles connected to conventional antibody production technologies is of enormous interest. To address this issue, we demonstrate the synthesis of complex antibody formats, in particular immunoglobulin G (IgG) and single-chain variable fragment Fc fusion (scFv-Fc), in a microsome-containing cell-free system based on translationally active chinese hamster ovary (CHO) cell lysates. To mimic the environment for antibody folding and assembly present in living cells, antibody genes were fused to an endoplasmic reticulum (ER)-specific signal sequence. Signal-peptide induced translocation of antibody polypeptide chains into the lumen of ER microsomes was found to be the prerequisite for antibody chain assembly and functionality. In this context, we show the rapid synthesis of antibody molecules in different reaction formats, including batch and continuous-exchange cell-free (CECF) reactions, depending on the amount of protein needed for further analysis. In addition, we demonstrate site-specific and residue-specific labeling of antibodies with fluorescent non-canonical amino acids. In summary, our study describes a novel antibody production platform which combines the highly efficient mammalian protein folding machinery of CHO cells with the benefits of cell-free protein synthesis.

## Introduction

Due to their remarkable abilities as binding and detection reagent, antibodies have become indispensable tools for biomedical applications including the treatment of cancer, autoimmune and inflammatory disorders^1–3^. Antibodies, or immunoglobulins, consist of several domains stabilized by intrachain disulfide bonds, whose quaternary structure is assembled by interchain disulfide bridges^4^. Immunoglobulin G, the antibody isotype most commonly used in diagnostics and therapeutics, is a heterotetramer composed of twelve domains within two identical heavy and two identical light polypeptide chains^5–8^.

Folding and assembly of antibody polypeptide chains takes place in the ER of B cells or plasma cells^9^. Due to its oxidative environment and the presence of specialized enzymes, such as protein disulfide isomerase (PDI), the ER provides optimal conditions for the formation of intra and interchain disulfide bonds^10^. In addition, ER-localized chaperones such as BiP (binding immunoglobulin protein) and enzymes like peptidyl-prolyl isomerase (PPI) or PDI are known to be essential for the folding and assembly of antibody molecules^11^. Apart from the formation of disulfide bonds and prolyl isomerization, antibodies are further modified by N-glycosylation in the Fc part of the heavy chain (HC) which is responsible for some effector functions and interactions with the immune system^12^. Due to this complex maturating process that antibodies undergo, it is not surprising that conventional antibody production technologies are based on mammalian expression systems, such as CHO cells. CHO cells are the most widely used expression host for recombinant therapeutic proteins with the majority of marketed antibodies being manufactured in this system^13,14^.

In the early phase of antibody development a multitude of different antibody variants has to be screened to find the optimal candidate for production. Typically, this screening procedure is facilitated by using transient cell-based expression technologies. Unfortunately, handling of mammalian cell cultures is laborious and time-consuming and can hardly be accelerated. Thus, we anticipate that a technology that is able to accelerate the antibody screening phase during lead identification and optimization will be highly in demand. To address this issue, we have developed a microsome-containing cell-free expression system based on CHO cells. The cell-free system developed combines the advantages of CHO cells as production host with the benefits of cell-free systems in general^15^. Originally, cell-free systems have been developed as a research tool to study the fundamentals of translation processes *in vitro* ^16^. Nowadays, cell-free systems have become a well-proven and economically beneficial alternative to conventional cell-based protein expression systems^17^. In cell-free systems, protein synthesis is disconnected from cell viability and fate since the reactions are usually conducted using thoroughly prepared cell lysates which contain the molecular components necessary for translation, such as ribosomes, translation factors and enzymes. To initiate protein synthesis, the cell lysate is mixed with template DNA or mRNA and supplemented with amino acids and energy-regenerating components. This easy-to-handle working process circumvents many of the issues connected with conventional cell-based expression platforms. For example, cell-free reactions can be performed in a flexible scale, from microliter to milliliter over liter scale, depending on the amount of protein needed for further analysis^18^. This easy adjustment of the manufacturing scale facilitates miniaturization, parallelization, and high-throughput analysis^19,20^. Moreover, the system easily allows interventions to optimize protein synthesis^21^ or folding^22^, since metabolic processes can be exclusively directed to the one target protein.

By using our CHO-based cell-free system antibody candidates can be synthesized in a very short time period in parallel reactions. In comparison to cell-based expression the cell-free system presented in this study offers several advantages: (i) By using the cell-free technology antibody candidates can be synthesized rapidly within a few hours of incubation time compared to weeks when using cell-based expression. Thus, information on the genetic design, expression titers and antigen binding properties is much faster accessible. (ii) Cell-free reactions can be operated in parallel which allows for the screening of hundreds of antibody candidates at once. (iii) Due to the open reaction design, cell-free systems can be easily manipulated, e.g. by the addition of non-canonical amino acids. The possibility to introduce non-canonical amino acids into *de novo* synthesized proteins allows for the synthesis and screening of site-specifically modified antibodies, which is an important issue in developing antibody-drug conjugates. (iv) By using cell-free systems, antibodies can be synthesized based on linear expression templates such as PCR fragments, an instance that circumvents time-consuming and labor-intensive cloning steps^23^.

The CHO cell-free system used in this study comprises endogenous microsomal vesicles which originate from the ER of the CHO cells used for lysate preparation. When fusing antibody gene templates to an appropriate signal sequence, *de novo* synthesized proteins can be translocated into ER derived microsomal vesicles where they find optimal conditions for folding and assembly thus mimicking the conditions for antibody folding and assembly as present in living cells. Until recently, microsome containing eukaryotic cell-free systems lagged behind prokaryotic ones when it came to production yields but have now caught up^24^. In this context, a high-yield cell-free system based on CHO cell lysates has been developed in our lab, demonstrating the synthesis of functionally active membrane proteins and also antibody fragments. Single-chain antibody fragments assemble from one polypeptide chain and typically require the formation of a maximum of two intramolecular disulfide bridges. In contrast, full length antibodies are much more complex and rely on the assembly of four separate polypeptide chains by intermolecular disulfide bonds in addition to the folding of each of the twelve or more individual immunoglobulin domains. In order to meet these challenges, we evaluated the use of our recently developed microsome-containing CHO cell-free system for the synthesis of complex antibody formats, in particular IgG and scFv-Fc. Furthermore, to mimic *in vivo* antibody folding in the cell-free system, DNA templates were fused to the ER-specific signal sequence of honeybee melittin, which has been shown before to allow for both efficient translation initiation and protein translocation into microsomal vesicles^24–27^. The developed cell-free system combines the advantages of the cell-free approach with the benefits of the highly efficient eukaryotic protein folding machinery of CHO cells. We anticipate that this system will constitute a novel tool for the acceleration of antibody development and screening during lead identification and optimization.

## Results

### IRES-mediated cell-free synthesis of different antibody constructs using the batch reaction format

**Figure 1:**
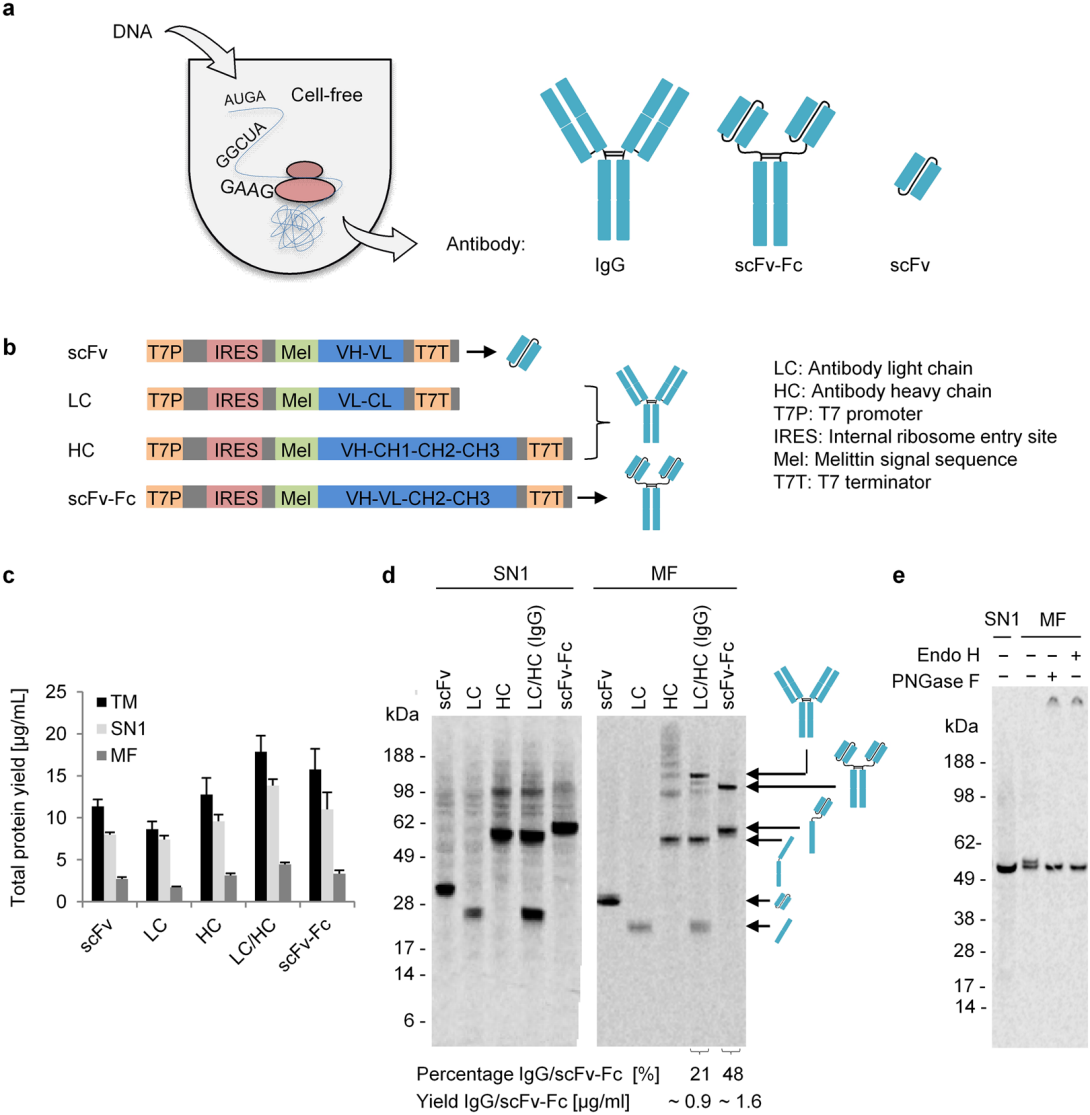
IRES-mediated cell-free synthesis of different antibody constructs (IgG, scFv-Fc, scFv) using the CHO cell-free system. (**a**) Schematic representation of the antibody formats synthesized in this study. (**b**) Schematic representation of the DNA template design applied in this study. Antibody light chain (LC) contains one variable domain (V_L_) and one constant domain (C_L_). Antibody heavy chain (HC) is composed of one variable domain (V_H_) and three constant domains (C_H_1, C_H_2, and C_H_3). (**c**) Diagram showing protein yields determined in the complete translation mixture (TM), the supernatant fraction after centrifugation (SN1) and the microsomal fraction (MF). Protein synthesis reactions were performed in the presence of ^14^C-leucine for subsequent qualitative and quantitative analysis. Standard deviations were calculated from triplicate analysis. (**d**) Qualitative analysis of cell-free synthesized proteins by SDS-PAGE and subsequent autoradiography. (**e**) Autoradiograph derived from SDS-PAGE gel showing glycosylation of HC in MF by digestion with EndoH and PNGase. scFv: single-chain variabe fragment; scFv-Fc: single-chain variable fragment Fc fusion.

We previously demonstrated the development and performance of a cell-free system based on translationally active CHO cell lysates^15,26–28^. The cell lysate used comprises endogenous microsomal vesicles having their origin in the ER. These vesicles allow a co-translational translocation of target proteins into their lumen and subsequent posttranslational modifications, such as the formation of disulfide bonds, which is one of the hallmarks of antibody domains and assembly. Translocation of *de novo* synthesized polypeptide chains was achieved by placing the melittin signal sequence upstream of the target gene (Fig. 1a,b). To test this configuration, three different antibody formats of the anti-SMAD2 antibody SH527-IIA^29,30^ (single chain variable fragment (scFv), scFv-Fc and IgG) were produced. First, protein synthesis reactions were performed using small-scale batch-based reactions in combination with circular DNA templates. By using this rapid high-throughput approach, total protein yields of 9 –18 µg/mL could be reached (Fig. 1c). Fractionation of translation mixtures (TM) by centrifugation revealed a protein distribution of 7–14 µg/mL in the supernatant (SN1) and 2–4 µg/mL in the microsomal fraction (MF). Although average protein yields in SN1 were higher compared to MF, distinct protein bands of assembled scFv-Fc and IgG molecules were only detectable in MF, but not in SN1 (Fig. 1d). Distinct and single protein bands of scFv and antibody light chain (LC) could be observed in the autoradiograph, whereas HC was detectable as monomer, as dimer and in multimeric form. Co-expression of LC and HC in a 1:1 molar ratio resulted in the detection of LC monomer (~26 kDa), HC monomer (~55 kDa) and the assembled LC/HC heterotetramer (IgG) showing an apparent molecular weight (MW) of approximately 160 kDa. scFv-Fc was detectable as monomer (~60 kDa), but also as homodimer with an apparent MW of 110 kDa. In order to estimate the yield of intact IgG and scFv-Fc, a densitometric analysis based on the obtained autoradiographs was performed. Under non-reducing conditions, approximately 21% intact IgG and 48% scFv-Fc could be detected, corresponding to 0.9 µg/ml IgG and 1.6 µg/ml scFv-Fc (Fig. 1d). As will be seen throughout all the presented data, the proportion of intact IgG and scFv-Fc varied between the different experiments ranging from 4–35% for IgG and 9–48% for scFv-Fc. By performing SDS-PAGE under reducing conditions HC dimers and multimers, scFv-Fc dimers and LC/HC heterotetramers were clearly identified as disulfide-bridged protein assemblies as their bands disappeared upon addition of the reducing agent DTT (Supplementary Fig. 1). In addition to the use of plasmids, linear DNA templates were applied for cell-free protein synthesis to underline the potential for high throughput applications. Using PCR products as template (Supplementary Fig. 2a), LC and HC were co-expressed using different template ratios in the cell-free reaction (LC/HC ratio 2:1, 1:1, 1:2, 1:3, 1:4). Protein synthesis based on linear DNA templates resulted in the assembly of IgG molecules as detected by autoradiography, identifying the PCR product ratio of 1:1 as the optimal one leading to the highest percentage of intact IgG (58%) (Supplementary Fig. 2b).

As reported in previous publications, microsome-containing lysates allow for the synthesis of N-glycosylated proteins^26,31^. Thus, glycosylation of HC molecules was analyzed by performing a deglycosylation assay using Peptide -*N*-Glycosidase F (PNGaseF) and Endoglycosidase H (EndoH). Without addition of endoglycosidases, a double band was observed in the autoradiograph, demonstrating the presence of two different protein species (Fig. 1e). Upon addition of either PNGaseF or EndoH, the upper protein band disappeared, which indicates that the band with the higher apparent MW was a result of glycosylation. Significantly, the double band was only detectable in MF, but not in SN1, indicating that glycosylation was only possible after translocation of target proteins into the lumen of microsomal vesicles.

**Figure 2:**
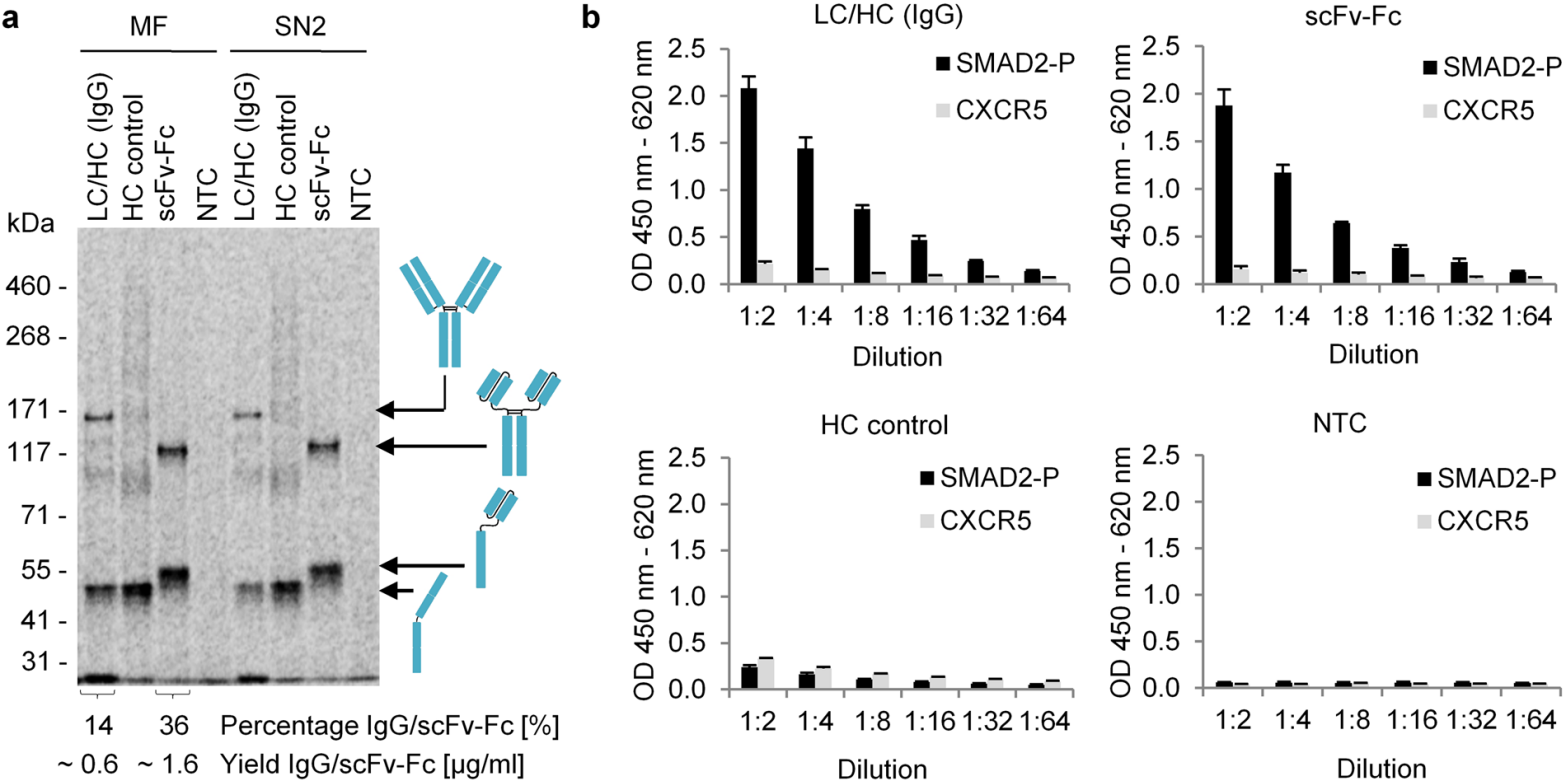
Functional analysis of cell-free synthesized antibody constructs by ELISA. (**a**) Autoradiograph derived from SDS-PAGE gel showing cell-free synthesized target proteins in the microsomal fraction (MF) and the supernatant fraction (SN2) after detergent-based release of target proteins from the lumen of microsomal vesicles. (**b**) ELISA analysis showing the specific binding of LC/HC (IgG) and scFv-Fc to its antigen SMAD2-P, but not to CXCR5 (non-related antigen). Samples analyzed by ELISA were treated likewise and tested in parallel in triplicate analysis. Starting concentrations in dilution 1:2 of LC/HC, scFv-Fc and HC control were 0.2 µg/mL each (total protein yield according to quantification via incorporation of ^14^C-leucine). Standard deviations were calculated from triplicate analysis. LC: antibody light chain; HC: antibody heavy chain; scFv-Fc: single-chain variable fragment Fc fusion; NTC: No template control.

To analyze synthesized antibody constructs for antigen-binding, translocated target proteins were released from the lumen of microsomal vesicles by detergent-containing buffer (see autoradiograph in Fig. 2a). Solubilized target proteins were separated from vesicular structures by another centrifugation step, resulting in lysate fraction SN2, which was directly applied for functional analysis by ELISA. Using a phosphorylated SMAD2 epitope peptide as antigen, strong binding was observed for the LC/HC co-expression as well as scFv-Fc samples, whereas both controls, the HC synthesized without addition of LC, and the translation mixture without addition of template (NTC), showed only background binding (Fig. 2b). To exclude that this binding was a result of unspecific stickiness, the non-related peptide antigen CXCR5 was analyzed in parallel. No binding was observed here. As an internal control, lysate fraction SN1 showing only trace levels of fully assembled antibody molecules was analyzed by ELISA. As expected, only weak antigen binding was observed for this lysate fraction (data not shown). As a consequence, functional analysis was performed using microsome-released antibody molecules present in lysate fraction SN2. Data obtained by ELISA is presented in Supplementary Fig. 3. To summarize, antibody molecules present in lysate fraction SN2 were found to be soluble, correctly assembled and functional in respect of antigen binding.

### Optimization of reaction conditions

Due to the open nature of cell-free reactions, synthesis conditions can be easily adjusted in order to achieve an optimal environment for protein synthesis, folding and assembly of multimeric proteins. Accordingly, we tested varying ratios of LC and HC template added to the cell-free reaction to increase the fraction of fully assembled IgG. This analysis has been based on the assumption that the ratio of the concentrations of the two templates directly influences reaction kinetics of transcription and translation. As described in literature, disulfide bonding between HC and LC is favored when LCs are present in folded state while HCs are only partly folded^32^ and a certain ratio of LC/HC expression was reported to be essential for successful production of IgG^33^. Based on these observations, HC and LC templates were either added simultaneously or LC template was added first, followed by the addition of HC template with a 15 min delay. Assembly of full length IgG molecules was monitored by autoradiography and functionality of cell-free synthesized antibodies was analyzed by ELISA. As can be seen in the autoradiograph in Supplementary Fig. 4, LC/HC heterotetramers were detectable in MF and SN2 with every ratio tested in both setups, simultaneous as well as time-delayed template addition (LC/HC ratios: 2:1, 1:1, 1:2, corresponding to template ratios of 60 nM: 30 nM, 30 nM: 30 nM, 30 nM: 60 nM, respectively). Amount of template added to the cell-free reaction corresponded to the detected band intensity, as HC and LC monomer bands were more pronounced depending on the total amount of the respective template added to the reaction. Differences in the fraction of assembled IgG, which could be detected in lysate fraction MF, were less pronounced after the detergent-based release to SN2. As protein yields were found to be comparable between the different reactions (Supplementary Fig. 4), samples were directly analyzed by ELISA. For the simultaneously added templates no significant differences were observed between the different applied template ratios (Supplementary Fig. 4). In contrast, the delayed addition of higher concentrations of HC template led to an increased ELISA signal (Supplementary Fig. 4), but despite this increase the overall activity of these samples was not significantly higher than the signals observed for the simultaneous template addition in ratio 1:1. Consequently, all of the following protein synthesis reactions were performed with a 1:1 ratio of simultaneously added LC/HC template.

**Figure 3:**
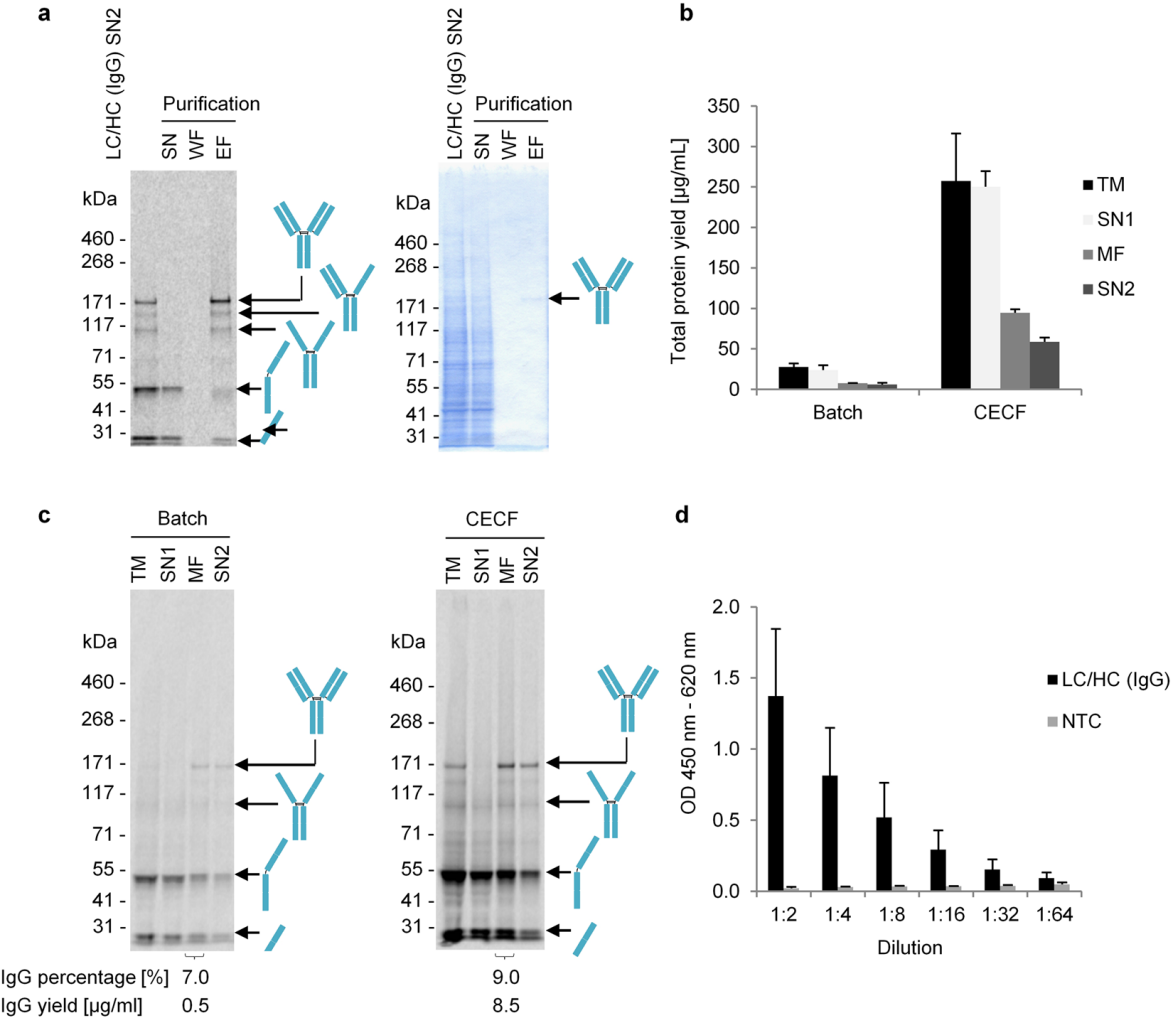
Comparison of batch- and CECF-based IRES-mediated cell-free synthesis of IgG. Protein synthesis reactions were performed in the presence of ^14^C-leucine for subsequent qualitative and quantitative analysis. (**a**) Autoradiograph derived from SDS-PAGE gel to monitor purification of IgG using Protein G coupled magnetic beads (batch-based synthesis). SN: bead supernatant, WF: washing fraction, EF: elution fraction. (**b**) Diagram showing protein yields determined in the complete translation mixture (TM), the supernatant fraction after centrifugation (SN1), microsomal fraction (MF) and supernatant fraction 2 after detergent solubilization (SN2). Standard deviations were calculated from triplicate analysis. (**c**) Qualitative analysis of cell-free synthesized proteins by SDS-PAGE and subsequent autoradiography. (**d**) ELISA analysis showing the specific binding of CECF-produced IgG to its antigen SMAD2-P. ELISA was performed with IgG present in SN2. Starting concentration in dilution 1:2 was approximatly 0.9 µg/mL total protein yield according to quantification via incorporation of ^14^C-leucine. Standard deviations were calculated from triplicate analysis. LC: antibody light chain; HC: antibody heavy chain; NTC: No template control.

As can be seen in the autoradiographs mentioned above, co-synthesis of LC and HC templates resulted in a mixture of proteins, namely LC and HC monomers, HC dimers as well as fully assembled IgG heterotetramers. In order to increase the fraction of fully assembled IgG, we evaluated additional experimental approaches such as evaluation of incubation time and temperature (Supplementary Fig. 5), co-expression of the chaperones BiP and PPI (Supplementary Fig. 6), as well as optimization of redox conditions by adding mixtures of reduced (GSH) and oxidized glutathione (GSSG) (Supplementary Fig. 7). Unexpectedly, these strategies did not significantly increase the amount of intact and functional IgG. In order to separate fully assembled IgG from unassembled antibody polypeptide chains, purification via commercially available magnetic beads coupled to Protein G was employed. Figure 3a shows that the fraction of full length IgG could be increased from approximately 14% (unpurified) to 54% upon a one-step purification. While the autoradiograph still shows trace levels of heterotrimers of one LC and two HC (~136 kDa), HC dimer (~110 kDa), as well as HC (~55 kDa) and LC monomers (~26 kDa), the coomassie stain of the corresponding SDS-PAGE-gel exclusively shows one defined band of full length antibody (~160 kDa). In general, Protein G purification resulted in a 3.3-fold increase (+/−0.55, value based on three independent experiments) of the fraction of fully assembled IgG compared to its side products (LC/HC monomers, HC dimers, 2xHC-1xLC trimers). Functionality of samples before and after purification was verified by ELISA, revealing decreased antigen binding properties of purified antibody samples compared to unpurified ones (Supplementary Fig. 8).

### Increasing protein yields of fully assembled IgG and scFv-Fc by changing the reaction format

The batch-based reaction format represents an easy-to-handle, fast and efficient way to assess the synthesis of a given target protein. Thus, it was chosen as method of choice to initially evaluate the cell-free synthesis of different antibody formats using CHO cell lysates. Protein yields that could be achieved with this method were sufficient to allow for a subsequent characterization of target proteins regarding their integrity, assembly to multimeric complexes and functionality, as described above. Nevertheless, higher protein yields may be required once certain antibody candidates are selected in a small-scale batch-based approach. As reported in literature, the CECF reaction mode is expected to significantly increase protein yields by prolonging the reaction life time^24,34,35^. We utilized this approach for the cell-free synthesis of scFv-Fc and IgG in a small-scale dialysis device, reaching total protein yields of approximately 250 µg/mL (LC/HC) (Fig. 3b) and 500 µg/mL (scFv-Fc) (Supplementary Fig. 9). In comparison to the corresponding batch-based reactions protein yields were increased 9-fold (IgG) and 11-fold (scFv-Fc) in TM or 12-fold (IgG) and 13-fold (scFv-Fc) in MF. Densitometric evaluation of the autoradiograph (Fig. 3c) indicated the following distribution of products: 22% LC (~20 µg/mL), 67% HC (~64 µg/mL), 2% HC dimer (~2 µg/mL) and 9% LC/HC heterotetramer (~9 µg/mL) (for calculation of band intensities see Supplementary Fig. 10). For scFv-Fc, 69% scFv-Fc monomer (~80 µg/mL) and 31% (~36 µg/mL) scFv-Fc dimer were determined (Supplementary Fig. 9). Based on the available data, no significant differences in the percentage of intact IgG and scFv-Fc were observed when changing from batch to CECF reactions. Nevertheless, the results show that the change from batch to CECF reactions increased total protein yields as well as the yield of intact scFv-Fc and IgG (~0.5 µg/ml IgG in batch vs. ~9 µg/mL IgG in CECF; ~0.8 µg/ml scFv-Fc in batch vs. ~36 µg/mL in CECF). Furthermore, corresponding batch and CECF samples were analyzed by ELISA showing the specific binding of CECF-produced IgG (Fig. 3d) and scFv-Fc (Supplementary Fig. 9) to its antigen SMAD2-P. Due to the detected increase in protein yields, subsequent experiments were performed using the CECF reaction mode.

### Labeling of antibody molecules by introduction of non-canonical amino acids

**Figure 4:**
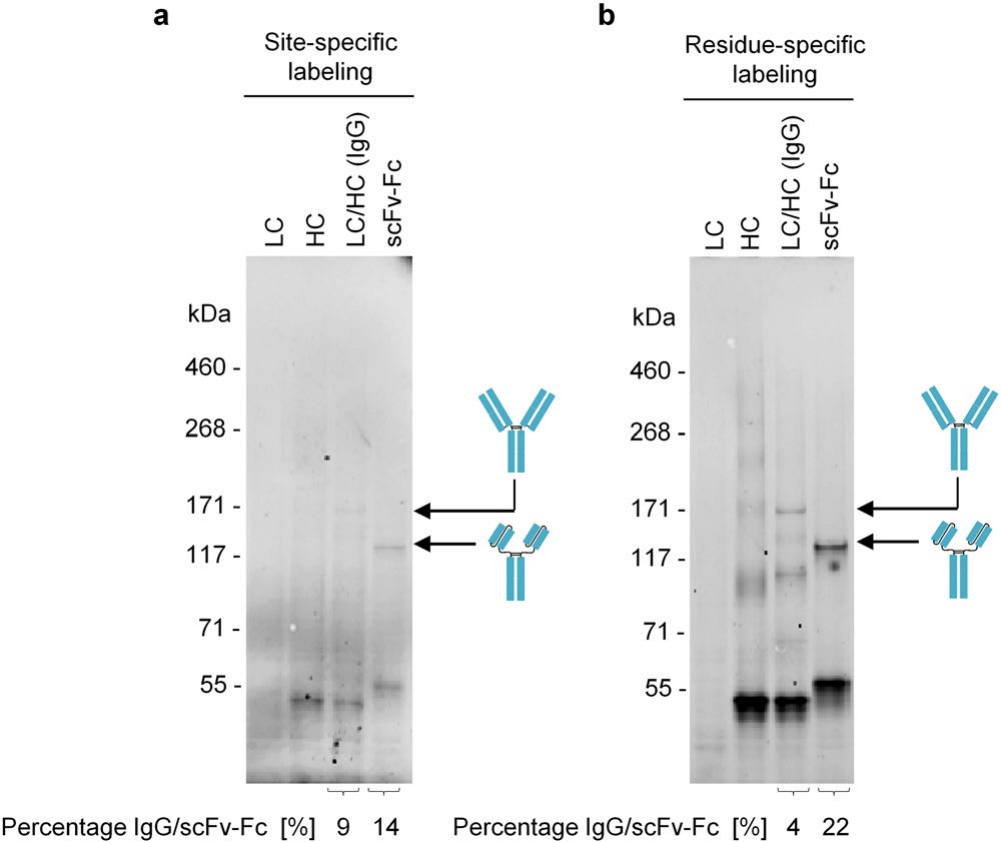
In-gel-fluorescence of cell-free produced IgG and scFv-Fc labeled with Bodipy-TMR-lysine. Labeling was achieved by supplementing the batch-based cell-free reaction either with BODIPY-TMR-lysine-tRNA_CUA_ (site-specific labeling) or BODIPY-TMR-lysine-tRNA_GAA_ (residue-specific labeling). (**a**) SDS-PAGE gel showing fluorescent protein bands of IgG and scFv-Fc in the microsomal fraction, site-specifically labeled at the position of the amber stop codon TAG located at the 3′-end of the open reading frame encoding HC as well as scFv-Fc template. (**b**) SDS-PAGE gel showing fluorescent protein bands of IgG and scFv-Fc (microsomal fraction) labeled by the incorporation of the fluorescent amino acid Bodipy-TMR-lysine at multiple sites in the protein (residue-specific labeling). LC: antibody light chain; HC: antibody heavy chain; scFv-Fc: single-chain variable fragment Fc fusion.

Further, we evaluated the possibility to label cell-free synthesized antibody molecules with non-canonical amino acids. Two approaches were applied: residue-specific labeling by sense codon reassignment and site-specific labeling by amber suppression. Site-specific labeling was achieved by supplementing the cell-free reaction with a suppressor tRNA (BODIPY-TMR-lysine-tRNA_CUA_) containing the anticodon complementary to the amber stop codon which has been introduced at the 3′ end of HC and scFv-Fc coding sequences (see Materials & Methods section “Template generation”). Using this approach, fluorescent protein bands of HC monomers (55 kDa), HC dimers (110 kDa) and LC/HC hetereotetramers (IgG) (160 kDa) could be observed directly by in-gel fluorescence. As expected, scFv-Fc could be detected as monomer (60 kDa) and as assembled dimer (120 kDa) (Fig. 4a). In addition, molecules were labeled in a residue-specific manner resulting in the introduction of non-canonical amino acids in a statistical way. As expected, protein bands appeared to be more intense compared to the site-specific labeling approach since several fluorescent amino acids can be introduced per antibody molecule (Fig. 4b).

### Functional characterization of full length IgG and scFv-Fc by surface plasmon resonance (SPR)

**Figure 5:**
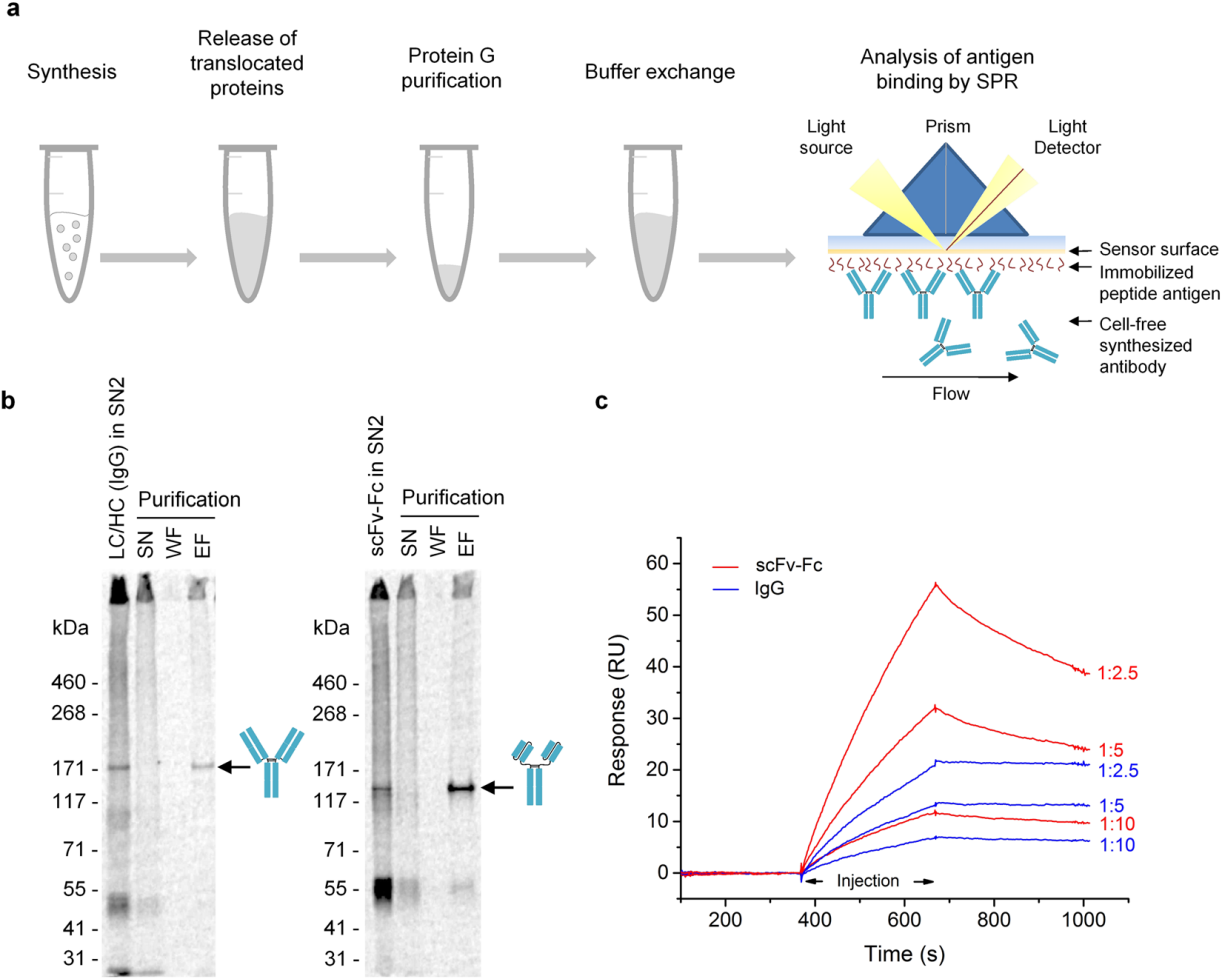
Analysis of CECF-produced IgG and scFv-Fc by surface plasmon resonance (SPR). (**a**) Scheme depicting the established work flow prior to SPR analysis. Sample preparation included cell-free synthesis of IgG and scFv-Fc in CECF reactions, release of translocated proteins from the lumen of microsomal vesicles, Protein G purification as well as desalting and buffer exchange, followed by subsequent SPR measurement of samples. (**b**) Autoradiograph derived from SDS-PAGE gel demonstrating purification of IgG (left) and scFv-Fc (right) from supernatant fraction 2 after detergent solubilization (SN2) using Protein G coupled magnetic beads. SN: bead supernatant, WF: washing fraction, EF: elution fraction. (**c**) Biacore sensorgrams showing the specific binding of purified scFv-Fc and IgG to immobilized peptide antigen SMAD2P. Samples were diluted 1:10, 1:5 and 1:2.5 to show concentration-dependent binding. Protein yields were assessed by the analysis of identically treated samples synthesized in the presence of ^14^C-leucine. Dilutions of 1:10, 1:5 and 1:2.5 correspond to protein concentrations of approximately 0.07 µg/mL, 0.15 µg/mL, 0.3 µg/mL containing Protein G purified IgG and 0.09 µg/mL, 0.18 µg/mL, 0.37 µg/mL containing Protein G purified scFv-Fc. Sensorgrams are double reference subtracted (CXCR5 as non-related peptide antigen, HC as analyte). LC: antibody light chain; HC: antibody heavy chain; scFv-Fc: single-chain variable fragment Fc fusion.

In addition to analysis by ELISA, antigen-binding properties of cell-free synthesized antibodies were evaluated by SPR. Prior to the measurement, biotinylated SMAD2-P peptide was immobilized onto the streptavidin coated sensor chip surface. Following cell-free protein synthesis (CECF, 24 h), microsomal vesicles were enriched by centrifugation and treated with detergent-containing buffer (0.2% n-Dodecyl β-D-maltoside (DDM) in PBS, 1 h) in order to release translocated and vesicle-trapped antibody molecules. This lysate fraction can be expected to contain not only *de novo* synthesized antibodies but also endogenous proteins which were solubilized by the detergent. Initial SPR measurements were performed by using this lysate fraction directly, but due to the presence of endogenous proteins, measurements were impaired by high unspecific signals. Thus, antibody samples were purified via protein G from the lysed vesicular preparation. Furthermore, a subsequent desalting step by size exclusion chromatography was done to remove low molecular weight components and thus to minimize bulk refractive index contributions (Fig. 5a and b). Injections of increasing concentrations of purified IgG and scFv-Fc (1:10, 1:5 and 1:2.5 dilutions) were conducted to verify concentration dependent association with their antigen SMAD2-P (Fig. 5b). The obtained binding curves show that increasing concentrations of IgG and scFv-Fc resulted in increased binding signals demonstrating the specificity of the binding (Fig. 5c). Interestingly, considerable differences between the binding behaviors of both antibody formats were detected: Concentration dependent measurements show an almost 3x times higher binding signal of scFv-Fc compared to IgG taking into account the different MW of both proteins. In addition, scFv-Fc dissociates more rapidly from the antigen than IgG (Fig. 5). Reasons for this observation are discussed below (discussion section).

**Figure 6:**
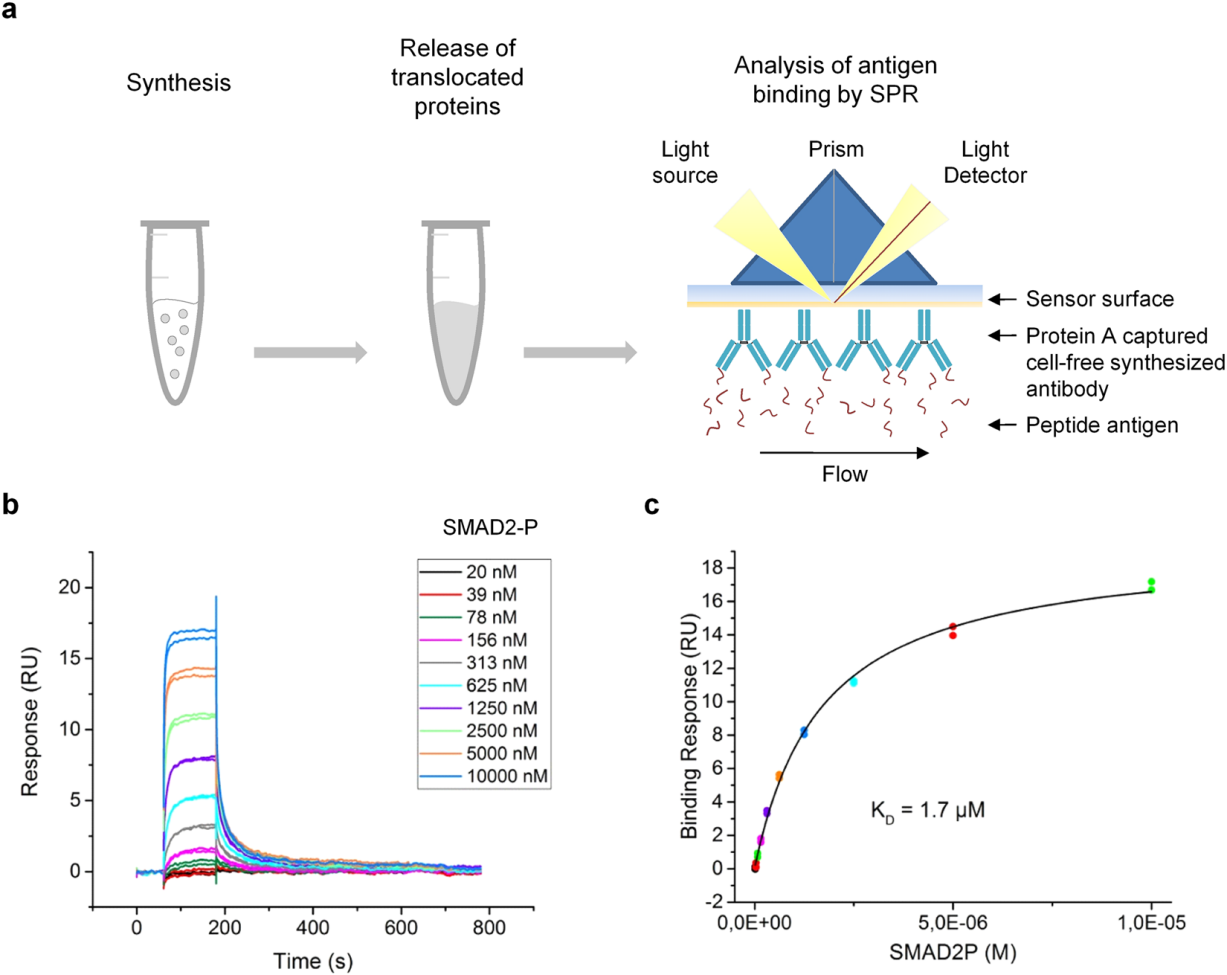
Steady-state affinity analysis of CECF-produced IgG. (**a**) Scheme depicting the applied work flow: Sample preparation included cell-free synthesis of IgG in CECF reactions and release of translocated proteins from the lumen of microsomal vesicles, followed by subsequent immobilization of antibodies to Protein A coated sensor chip. (**b**) Overlay of Biacore sensorgrams from two consecutive experiments showing the binding of peptide antigen SMAD2-P to immobilized IgG. Peptide antigen SMAD2-P was injected with increasing concentrations to determine the affinity in steady-state. Sensorgrams are reference subtracted (HC). (**c**) Binding curve derived from b confirming the functional activity of cell-free produced IgG with a K_D_ of 1.7 µM.

The SPR analysis (Fig. 5c) revealed concentration-dependent binding of cell-free synthesized antibody molecules, while an exact calculation of the kinetic parameters was not possible as (1) no regeneration of the chip surface was performed to avoid possible negative influences of the regeneration solution, (2) applied sample concentrations were too low and (3) a bivalent binding behavior was expected. Due to these constraints, dissociation equilibrium constants (K_D_) were determined by applying the reverse approach, binding of peptides to sensor chip immobilized IgG (Fig. 6a). Figure 6b shows the expected binding behavior: Injections of increasing concentrations of peptide result in an increase in binding signal. Plotting of signals at the end of the association phase against peptide concentration and fitting of data using the steady-state affinity model, revealed a K_D_ value of 1.7 µM (*R*_*max*_ = 19.4 RU; *chi* ^*2*^ = 0.102) of cell-free synthesized, SMAD2-P specific IgG (Fig. 6c). Besides analysis of data using the preferred steady-state affinity model, additional fittings (“1:1 kinetic” and “heterogeneous ligand”) were conducted which led to comparable results underlining the revealed K_D_ value of 1.7 µM (Supplementary Fig. 11).

## Discussion

In this study, we demonstrate the synthesis of three different, commonly used antibody formats (IgG, scFv-Fc and scFv) in a microsome-containing cell-free system based on CHO cell lysates. To our knowledge this is the first study showing the synthesis of functional full length antibodies in a microsome containing CHO cell-free system. Using batch-based reactions, antibodies were rapidly synthesized within three hours of incubation with predictable yields. All components necessary for cell-free protein synthesis could be combined in three freeze-thaw stable mixes (A, B, C) which contributes to the ease, speed and reliability of the system applied.

A prerequisite for IgG functionality is the assembly of two heavy and two light polypeptide chains into a defined quaternary structure^32^. As expected, fully assembled IgG and thus functional antibodies were only detected upon signal peptide-induced translocation into ER-derived microsomal vesicles. In addition, a major fraction of antibody molecules was detected in lysate fraction SN1, representing the non-translocated protein fraction. Proteins found in SN1 were soluble, but non-functional, since no dimer (scFv-Fc) or heterotetramer formation (IgG) was observed. The redox potential in cell lysates can be efficiently tuned by supplementing reactions with mixtures of GSH and GSSG^36–38^. Unexpectedly, supplementation of the CHO cell-free system with GSH/GSSG did not increase the level of assembled antibody molecules in SN1 (Supplementary Fig. 7). Effects of this supplementation were only visible in the microsomal fraction (MF), resulting in a higher proportion of assembled IgG, but overall more GSSG decreased protein yields in the reaction. Consequently, this approach has not been followed further. For the future, it seems to be reasonable to change the applied insect signal sequence to one of mammalian origin which might enhance translocation efficiency into microsomal vesicles. Optimizing signal peptides to improve antibody secretion efficiency is a promising strategy as has been already shown in *in vivo* experiments^39^. Nevertheless, it has to be noted that also other reasons might contribute to the relatively small fraction of translocated compared to non-translocated proteins. For example, centrifugation of CHO microsomes at 16,000 × g for 10 min might not result in a quantitative pelletization of microsomes and thus translocated proteins. In addition, the high productivity of the cell-free system due to efficient IRES mediated translation initiation may be just capped by the availability of the translocation machinery present in the system. For example, a limitation of signal recognition particles which mediate the transport of ribosome, mRNA, and nascent polypeptide chain would also explain the distribution of translocated versus non-translocated proteins. Indications for such a limitation have already been shown in *in vivo* studies underlining this assumption^40^. Even though total protein yields determined in MF were lower compared to SN1, obtained yields were found to be sufficient for subsequent quantitative and qualitative analysis by ELISA. In addition, we observed that only a fraction of *de novo* synthesized protein detected in MF accounted for intact IgG/scFv-Fc. In average 15% (+/−10, value based on nine independent experiments) assembled IgG and 27% (+/−14.4, value based on six independent experiments) scFv-Fc were detected in the microsomal fraction, but it has to be noted that these values varied notably between different experiments.

The reasons for these observations yet remain unclear. Future examination of various antibody candidates might be necessary to assess if this is a general attribute of the system or refers to the particular antibody analyzed in this study. The switch from batch to CECF reactions did not increase the proportion of intact IgG notably (7% in batch vs. 9% in CECF), but with the gain of total protein yield the amount of intact protein increased as well (Fig. 3). In contrast, for scFv-Fc 9.3% assembled dimer were detected after synthesis in batch compared to 30.8% in CECF reaction. However, the ratio of intact scFv-Fc varied between the different experiments as a maximum of 48% intact scFv-Fc was already obtained in a batch reaction (Fig. 1). In order to enhance the assembly of intact antibody heterotetramers, one can also consider treating the microsomes *after* completing the protein synthesis reaction with an oxidizing buffer. The success of this strategy has been shown before^36^ and our preliminary data, where we use this strategy to promote disulfide bond formation and thus to increase the proportion of intact IgG, looks promising as well (Supplementary Fig. 12). Nevertheless, further optimization of the reaction conditions during protein synthesis and afterwards is necessary to overcome the limitation of the observed partial protein assembly in CHO microsomes.

Interestingly, SPR measurements revealed considerable differences between the binding behaviors of IgG and scFv-Fc: In concentration dependent measurements scFv-Fc showed an almost 3x times higher binding signal compared to IgG, but scFv-Fc dissociated more rapidly from the antigen than IgG (Fig. 5). Reasons for this observation can be manifold: For example, the observed binding behavior may indicate a larger percentage of functional scFv-Fc in the sample compared to IgG, for example due to differences in the efficiency of Protein G purification. Also, structural differences between IgG and scFv-Fc might lead to the differences in binding behavior. V_H_ and V_L_ domains of scFv-Fc are covalently connected by a peptide linker, while V_H_ and V_L_ of IgG are connected by non-covalent interactions in addition to the disulfide bridge connecting C_L_ and C_H_1. In this context, it has been reported that the conversion of scFv antibody fragments to full length IgG can lead to an increase, a decrease or even to a complete loss in functionality^41–43^. So far, the original phage display derived recombinant human antibody SH527-IIA4^44,45^ has not been characterized with respect to kinetic binding parameters. In this study, a K_D_ value for the cell-free synthesized IgG of 1.7 µM was determined. This value shows that the affinity of the antibody SH527-IIA4 lies in an acceptable, though not excellent range.

So far, full length IgG molecules have been produced in cell-free systems based on *Escherichia coli* (*E. coli*)^46,47^, tobacco BY-2 cell lysates^48^ and very recently also in a CHO-based cell-free system^49^. Antibody synthesis in *E. coli* based cell-free systems has made remarkable advances over the last decade, starting from yields of 1 µg/mL total antibody^47^ up to several hundred micrograms per milliliter reaction volume^46^. A huge amount of effort was put into the optimization of reaction conditions to achieve a cell-free environment which favors disulfide bonding and thus antibody chain assembly and folding^50^. Generally, these systems needed to be supplemented with chaperones and enzymes in order to reach high titers of functional antibodies. Despite these remarkable advances, it remains to be seen if prokaryotic cell-based systems and prokaryotic cell-free systems in particular are able to compete with conventional antibody production strategies using mammalian cells. Issues such as the lack of glycosylation and the danger of endotoxin contamination are still present and limit the use of *E. coli* produced antibodies in preclinical and clinical applications.

An advantage of the expression platform described in this publication is its close relation to fermentation in CHO cells, the most commonly used *in vivo* strategy applied for therapeutic antibody production. The strong interest in CHO based cell-free expression platforms is also visible in two recently published papers which present the synthesis of GFP^51^ and monoclonal antibodies^49^ using CHO lysates. In the latter publication, synthesis is based on the commercially available “1-Step CHO High-Yield IVT Kit” (Thermo Scientific, West Palm Beach, FL) which has been extensively optimized to allow for the synthesis of active, aglycosylated antibodies. As has been shown by Martin *et al*., reactions needed to be supplemented with GSSG/GSH in order to achieve fully assembled antibodies. A further optimization of the system with PDI, DsbC and by varying the template ratio could increase the percentage of intact antibody up to 15%^49^.

Despite the use of CHO lysates in general, the cell-free system described here offers the advantage of synthesizing antibodies in the presence of ER-derived microsomal vesicles which mimic the environment wherein antibodies fold *in vivo*. This brings the benefit that the enzymes and chaperones necessary for antibody folding and assembly are expected to be already present in the system. Interestingly, cell-free co-expression of the chaperones BiP and PPI did not enhance antibody folding and functionality, assuming that cell-free synthesized chaperones were not active or the concentration of endogenously contained folding helpers was not the limiting factor for correct antibody assembly. Antibodies that have been translocated into the lumen of these vesicles can be easily separated from the translation mixture by centrifugation, making subsequent purification steps easier. In addition, compared to other systems the possibility to synthesize glycosylated proteins in a cell-free system is advantageous as antibody characteristics and action may change upon glycosylation. Antibodies are glycosylated at an asparagine residue found within the Fc region. The glycosylation machinery in microsomal vesicles seemed to occur, while not 100% efficient, as demonstrated by the size change after digestion with endoglycosidases. As the microsomal vesicles are ER-derived, glycosylation in the system is expected to be exclusively N-glycosylation. Furthermore, it is expected that the complexity and trimming of sugar moieties is also limited to the enzymatic processes happening at the level of the ER. The observed sensitivity of the glycan structure for Endo H is supporting this assumption. It is known that glycoproteins are sensitive for Endo H as long as their glycosylation is on the level of the ER and the early regions of the Golgi complex^52^. Therefore, our results indicate that the glycan structure belongs to the class of high mannose glycan structure found on the level of the ER. While *E. coli* based systems do not provide glycosylation at all, the function of the glycosylation forms observed in this study in respect of ADCC or CDC remains to be confirmed. In this context it will be necessary to choose antibody candidates as models which bind to cell surface expressed targets.

Antibody-drug-conjugates (ADCs) represent a novel and promising class of oncology therapeutics, which facilitate the targeting of cytotoxic payloads to cancer cells, ideally leading to reduced side effects and a greater therapeutic window of the chemotherapeutic reagent^53^. One possibility to obtain conjugated proteins, such as ADCs, is to modify target proteins by the introduction of non-canonical amino acids. As has been already demonstrated in *E. coli* ^54–56^ and also eukaryotic cell-free systems^24,57^, stop codon suppression represents an elegant way to label proteins at one exactly defined position, thus avoiding product inhomogeneity or unwanted negative effects of multiple labels on antibody functionality. This opens up new strategies for the generation of ADCs. To show the proof-of-concept, the antibody molecules described in this publication were labeled with fluorescent amino acids. While two different labeling strategies have been applied, site-specific labeling is certainly the preferred one when it comes to the generation of ADCs.

In addition to this, the cell-free approach described here may also provide new opportunities for the initial screening and candidate selection of bispecific antibodies. In this context, the knobs-into-hole (KIH) strategy has emerged as method of choice for the generation of many bispecific IgG-like structures^58,59^. Open cell-free systems can provide a valuable complement to cell-based expression, since template ratios can be extensively optimized without the constraints of a cell membrane, and thus the necessity of plasmid transduction^58,60^. Using eukaryotic cell-free systems for candidate screening in the early antibody development phase might bring the additional advantage of being close to the final production system - which are in most cases CHO cells anyway.

In summary, we demonstrate a novel antibody screening technology based on a microsome-containing CHO cell-free system which combines the advantages of the eukaryotic expression host with the benefits of cell-free systems in general. The CHO cell-free system described in this publication provides a new platform technology that can be very helpful in the early antibody development phase where multiple antibody candidates, templates and sequences need to be screened in parallel. Both cell-free reaction formats applied in this study, batch and CECF, are amenable for high-throughput applications, as has been shown in previous publications^20,61,62^. In comparison to candidate selection in mammalian cell culture this cell-free system may dramatically simplify and accelerate routine lab work. Moreover, the system may provide new opportunities for the rapid screening and development of ADCs by site-specific incorporation of non-canonical amino acids as well as alternative antibody formats such as bispecific antibodies.

## Methods

### Template generation

The recombinant human antibody SH527-IIA4 against SMAD2-P (where SMAD is “mothers against decapentaplegic homolog 2″ and SMAD2-P is phosphorylated SMAD2 peptide) was selected by phage display using the human naive antibody gene library HAL7/8^44^ as described before^45^.

Based on the sequence information of the variable domains of the light and heavy antibody chains, three different antibody constructs were designed: 1) single-chain variable fragment (scFv) (pIX3.0-scFv-IIA4-His-c-Myc, 3936 bp), 2) single-chain variable fragment Fc fusion (scFv-Fc) (pMA-scFv-Fc-IIA4-His-c-Myc-amb, 4382 bp) and 3) full length IgG consisting of antibody LC (VL-CL) (pMA-LC-IIA4, 3440 bp) and antibody HC (VH-CH1-CH2-CH3) (pMA-HC-IIA4-His-c-Myc-amb, 4267 bp). Coding sequences of antibody genes were fused N-terminally to the melittin signal sequence to translocate *de novo* synthesized polypeptide chains into the lumen of the microsomal vesicles. HC was furthermore equipped with a C-terminal His-Tag and c-myc-Tag sequence followed by an amber stop codon to allow for the site-specific introduction of non-canonical amino acids. The 5′ untranslated region (5′UTR) of each construct contained regulatory sequences such as the T7 promotor sequence and an internal ribosomal entry site (IRES from the intergenic region (IGR) of the Cricket paralysis virus (CrPV)) to allow for efficient transcription based on T7 RNA polymerase and initiation factor-independent translation initiation, respectively^26^. All templates used in this study were equipped with identical 5′ and 3′ UTRs. Constructs based on the pMA vector backbone were synthesized *de novo* by Geneart (Life technologies, Thermo Fisher). Plasmid pIX3.0-scFv-IIA4-His-c-Myc was generated as follows: Initially, scFv gene and CrPV IRES were amplified separately by PCR using the following gene-specific primers: X-Mel-anti-SH527-IIA4-F (5′-TACATTTCTTACATCTATGCGGACCAGGTGCAGCTGCA GGAGT-3′) and C-pHAL14-R (5′-TCTTGGTTAGTTAGTTATTAA TTCAGATCCTCTTCTGAGAT-3′) using plasmid pHAL14-SH527-IIA4 (Technische Universität Braunschweig, Prof. Michael Hust) as template for scFv amplification and XF (5′-ATGATATCTCGAGCGGCCGCTAGCTAATACGACTCACTATAG-3′) and Mel-R (5′-GTCCGCATAGATGTAAGAAATG-3′) using pIX3.0-NCM-Nluc as template for IGR IRES amplification. After purification by size exclusion chromatography (QIAquick-PCR Purification Kit, Qiagen), PCR products were mixed in an equimolar ratio and fused by overlap-extension PCR using the primers XF and XR (5′-ATGATATCACCGGTGAATTCGGATCCAAAAAACCCCTCAAGAC-3′). Subsequently, purified fusion constructs were digested with *Xho*I and *Eco*RI (New England Biolabs) and ligated into the linearized and dephosphorylated vector backbone pIX3.0 (Qiagen). The integrity of generated constructs was verified by restriction digestion, agarose gel electrophoresis and sequencing. Plasmids suitable for cell-free protein synthesis were prepared by using the PureLink^®^HiPure Plasmid Midiprep Kit (Thermo Fisher) according to the manufacturer’s instructions. PCR products of LC and HC were generated based on plasmid templates pMA-LC-IIA4 and pMA-HC-IIA4-His-c-Myc-amb by using XF and XR as primers for amplification. PCR reactions were purified by size exclusion chromatography (Invisorb^®^ Fragment CleanUp) to desalt the sample and subsequently concentrated by using a centrifugal evaporator (Eppendorf Concentrator 5301). Calculated fragment sizes were 1099 bp (LC) and 1897 bp (HC).

### Cell-free protein synthesis

Cell-free protein synthesis was performed based on translationally active CHO lysates as described previously^26,27^. In brief, batch-based cell-free reactions were composed of three different premixes (A, B, C), containing the components necessary for *in vitro* transcription and translation. Premix A (10x) was composed of 300 mM HEPES-KOH (pH 7.6), 2250 mM KOAc, 2.5 mM spermidine, 1 mM of the 20 standard amino acids each (Merck) and 39 mM Mg(OAc)_2_. Premix B (2.5x) contained the S7 nuclease-treated CHO lysate supplemented with 250 µg/mL creatine kinase (Roche) and 50 µg/mL bulk yeast tRNA (Roche) (f.c. 40% CHO lysate in the reaction). CHO lysates were prepared from cultured CHO K1 cells as described previously^15,27^. Premix C (5x) consisted of 100 mM creatine phosphate, 8.75 mM ATP, 1.5 mM CTP, 1.5 mM UTP, 1.5 mM GTP (Roche) and 1.65 mM m^7^G(ppp)G cap analogue (Prof. Edward Darzynkiewicz, Warsaw University, Poland). Small scale, batch-based cell-free reactions were composed of premix A, premix B and premix C diluted in RNase-free water to result in 1x concentrated solutions and1 U/µL (f.c.) T7 RNA polymerase (Agilent). Template DNA was added at a final concentration of 60 nM.

Continuous-exchange cell-free reactions were performed using a commercially available two-chamber dialysis device (5PRIME) consisting of a 50 µL reaction and a 1,000 µL feeding chamber which are separated by a semipermeable dialysis membrane (cut-off 10 kDa). Two mixes, the reaction and the feeding mixture, were prepared separately from each other and filled into the appropriate chambers of the device. The reaction mixture (total volume 50 µL) was composed of premix A, premix B and premix D, diluted in RNase-free water to result in 1x concentrated solutions, 1 U/µL (f.c.) T7 RNA polymerase (Agilent), 0.02% (f.c.) sodium azide and 3.7 mM (f.c.) extra Mg(OAc)_2_. Template DNA was added at a final concentration of 60 nM. Premix A and B were composed as described above. Premix D (5x) consisted of 100 mM creatine phosphate, 8.75 mM ATP, 1.5 mM CTP, 1.5 mM UTP and 1.5 mM GTP (Roche). The feeding mixture (total volume 1,000 µL) was composed of 100 µL premix A (10x), 200 µL premix D (5x) and 0.02% (f.c.) sodium azide. Reactions were carried out at 27 °C for 24 h and 600 rpm.

For subsequent qualitative and quantitative analysis by autoradiography and liquid scintillation counting, cell-free reactions (batch) and feeding mixtures (CECF) were supplemented with ^14^C-leucine at 11.2–50 µM (specific radioactivity 10–66.67 dpm/pmol, Perkin Elmer).

### Determination of total protein yields

Yields of ^14^C-leucine labeled proteins were determined in TM, SN1 after centrifugation (16,000 × g, 10 min, 4 °C) and MF after resuspension of microsomes in phosphate-buffered saline (PBS, pH 7.4). To release translocated target proteins from the lumen of the microsomal vesicles, microsomes were resuspended in detergent containing buffer (0.2% DDM in PBS) and incubated under rigorous mixing for 45 min at RT. Subsequently, solutions were centrifuged and the resulting supernatant (SN2) was applied for further analysis. Following cell-free protein synthesis and fractionation into SN1, MF and SN2, aliquots of 3 µL were withdrawn from the solution, mixed with 3 mL trichloroacetic acid (TCA) and incubated in a 80 °C water bath for 15 min, followed by incubation on ice for 30 min. In order to remove non-incorporated ^14^C-leucine, protein solutions were filtered using a vacuum filtration system (Hoefer). Incorporation of ^14^C-leucine in cell-free expressed proteins was measured by liquid scintillation counting using the LS6500 Multi-Purpose scintillation counter (Beckman Coulter).

### SDS-PAGE, in-gel fluorescence, western blot and autoradiography

Sodium dodecyl sulfate polyacrylamide gel electrophoresis (SDS-PAGE) was performed using precast gels (NuPAGE, 10% Bis-Tris and 3–8% Tris-Acetate precast gels, Life technologies) under non-reducing conditions to preserve disulfide bond formation. In general, 3 µL aliquots of TM, SN1, MF and SN2 were mixed with 9 µL 1.3x sample buffer (NuPAGE LDS Sample Buffer, Life technologies) and incubated for 15 min at RT under gently shaking. Subsequently, samples were heated for 10 min at 70 °C, loaded onto SDS-PAGE gels and run for 35 min at 200 V (10% Bis-Tris gels) or 60 min at 150 V (3–8% Tris-Acetate gels). Following electrophoresis, gels were stained with Coomassie Blue (SimplyBlue SafeStain, Life technologies). After staining, gels were dried on Whatman paper for 60 min at 70 °C (Unigeldryer 3545D, Uniequip) and radioactively labeled proteins were visualized using a phosphorimager system (Typhoon TRIO + Imager, GE Healthcare). To estimate the percentage of intact IgG and scFv-Fc, autoradiograms were analyzed by densitometry using ImageQuantTL software. Residue- and site-specific labeling of antibody constructs was achieved by supplementing cell-free reactions with BODIPY-TMR-lysine-tRNA_GAA_ (f.c. 2 µM) and BODIPY-TMR-lysine-tRNA_CUA_ (f.c. 5 µM), respectively, which were added to the translation mixture 10 min after starting the reaction. Using the residue-specific labeling technique, the fluorescent amino acid is incorporated at multiple sites in the protein. Based on its anticodon, the pre-charged tRNA decodes the TTC codon, resulting in a competition of endogenous Phe-tRNA_GAA_ with pre-charged tRNAs. This leads to the incorporation of the fluorescent amino acid in a statistical manner. The incorporation of fluorescent amino acids in a site-directed manner was performed by using DNA templates bearing the amber stop codon TAG at the 3′-end of the open reading frame encoding HC template. Labelled antibody constructs were separated on 3–8% Tris-Acetate gels as described above. After electrophoresis, gels were incubated in a 50% methanol/dH_2_O solution. Labeled proteins were visualized by in-gel fluorescence using a phosphorimager system (excitation 532 nm, emission filter 580 nm band-pass, Typhoon TRIO + Imager, GE Healthcare).

Western blots were performed using the “iBlot Gel Transfer Device” (Life Technologies) according to the manufacturer’s instructions. In this way, proteins separated by SDS-PAGE were transferred onto a PVDF membrane. Blotting was performed using program number 3 (20 V, 10 min). Western blot membranes were blocked with 2% BSA in TBST (TBS containing 0.1% Tween-20). Subsequently, membranes were incubated with “Mouse anti-c-Myc unconjugated monoclonal antibody”, diluted in 1:100 in 1% BSA in TBST, overnight at 4 °C. “Anti-mouse-IgG-HRP linked AB”, diluted in 1:100 in 1% BSA in TBST, was used as secondary antibody and incubated for 1.5 h at RT. After each incubation step western blot membranes were washed 3x with TBST. Detection of protein bands was achieved by applying the “Amersham ECL Select Western Blotting Detection Reagent” (GE Healthcare) and measuring chemiluminescence by using “Typhoon Trio + Variable Mode Imager” (GE Healthcare). Following the measurement, membranes were washed with TBST, dried and subjected to autoradiographical analysis.

### Endoglycosidase and Proteinase K digestion

To analyze glycosylation of *de novo* synthesized proteins, deglycosylation assays using either EndoH or PNGaseF (NEB) were performed according to the manufacturer’s instructions. Aliquots of 5 µL of MF were treated with either EndoH or PNGaseF, followed by SDS-PAGE (NuPAGE, Novex™ 4–12% Bis-Tris Protein precast gels, Life technologies) and autoradiography as described above.

### Affinity purification

Purification of cell-free synthesized antibody constructs from lysate fraction SN2 was performed by using the “Immunoprecipitation Kit - Dynabeads^®^ Protein G” (Invitrogen) according to the manufacturer’s instructions. Elution fractions of 20 µL each were immediately neutralized by adding 2 µL of 1.5 M Tris (pH 8.8) buffer and frozen at −20 °C until further analysis.

### ELISA

Functional analysis of cell-free synthesized antibody fragments by ELISA was performed as described previously^29^. In brief, 96-well microtitre plates (Corning) were coated with streptavidin (f.c. 0.74 µg/mL in PBS, 270 µL/well) for 1 h at RT. This step was followed by 2x washing with dH_2_O containing 0.05% Tween-20. Blocking was performed by incubating wells with 2% BSA in PBS (270 µL/well) over night at 4 °C. After washing 3x with dH_2_O containing 0.05% Tween-20, wells were coated with biotinylated antigens (f.c. 2 µg/mL, 100 µL/well) for 1 h at RT. The following biotinylated peptides were used as antigens in this study: phosphorylated SMAD2 peptide (SMAD2-P: Biotin-PEG-(GGS)_2_GPLQWLDKVLTQMGSPSVRCSpSMpS) (where SMAD is “mothers against decapentaplegic homolog 2″) and CXC motivchemokin receptor 5 (CXCR5: Biotin-PEG-(GGS)_2_SLVENHLCPATEGPLMASFKAVFVP) peptide. Peptides were purchased from Peps4LS GmbH. Following 3x washing with dH_2_O containing 0.05% Tween-20, wells were incubated for 1.5 h at RT with cell-free synthesized antibody constructs contained in lysate fraction SN2. SN2 was tested in serial dilutions (1:2, 1:4, 1:8, 1:16, 1:32 and 1:64) using 1% BSA in PBS containing 0.05% Tween-20 (hereinafter referred to as PBST, pH 7.4) as dilution buffer. As a control, SN2 gained from translation mixture without addition of a DNA template (NTC) was tested in parallel. SN2 fractions were incubated for 1.5 h at RT. After washing the plates with PBST 3x, wells were incubated with the monoclonal anti-c-Myc-tag antibody 9E10 (dilution 1:1,000 in 1% BSA in PBST, 100 µL/well) for 1.5 h at RT. Then, wells were washed (3x in PBST) and incubated with a secondary anti-mouse-IgG HRP-linked antibody (dilution 1:2,000 in 1% BSA in PBST, 100 µL/well). After washing the wells 3x with PBST, detection of bound secondary antibodies was performed by adding TMB substrate solution (Life technologies). Color development was stopped after an incubation time of approximately 15 min by addition of 0.5 M H_2_SO_4_ (100 µL/well). Absorbance was measured at 450 nm (reference 620 nm) using the FLUOstar Omega (BMG Labtech).

### Surface plasmon resonance (SPR)

SPR binding experiments were performed on a Biacore™ T200 (GE-Healthcare Bio-Sciences AB). To analyze concentration-dependent binding of cell-free synthesized IgG and scFv-Fc, biotinylated peptide antigen SMAD2-P (0.1 µM in PBST) and CXCR5 (non-related antigen) (0.1 µM in PBST) were immobilized on streptavidin-coated sensor chips (Series S Sensor Chip SA, GE Healthcare). The following conditions were used for binding experiments: Flow cell temperature 25 °C, running buffer PBST and flow rate of 10 µL/min. Prior to the measurement, cell-free synthesized antibodies present in lysate fraction SN2 were purified using Protein G coupled magnetic beads (Immunoprecipitation Kit - Dynabeads^®^ Protein G, Invitrogen) to reduce non-specific binding to the sensor chip surface. Additionally, samples were subsequently purified using size exclusion chromatography (Zeba™ Spin Desalting Columns, 40 K MWCO, 0.5 mL, Thermo Fisher Scientific) to minimize bulk refractive index differences. Measurements were performed by injecting serial dilutions of purified samples for 300 s followed by dissociation for 300 s (flow rate 10 µL/min). Intermediate regeneration steps were not performed to avoid possible negative influences caused by the regeneration solution. Samples were injected in the following order: 1:10 IgG, HC and scFv-Fc, followed by 1:5 and 1:2.5 dilutions of each sample. Protein yields were assessed by the analysis of identically treated samples synthesized in the presence of ^14^C-leucine. Dilutions of 1:10, 1:5 and 1:2.5 correspond to protein concentrations of approximately 0.07 µg/mL, 0.15 µg/mL, 0.3 µg/mL containing Protein G purified IgG and 0.09 µg/mL, 0.18 µg/mL, 0.37 µg/mL containing Protein G purified scFv-Fc. As internal controls, translation mixtures containing no DNA template (NTC) or HC template alone (without LC) were treated identically and measured in parallel to monitor background binding. As antigen binding is dependent on the formation of the antigen-binding region consisting of the variable domains of LC and HC, synthesis of HC monomers and dimers alone was not expected to result in antigen-binding proteins. Binding signals were evaluated by using the Biacore T200 evaluation software (version 2.0 and BIAevaluation software version 3.01) and sensorgrams were double reference substracted (CXCR5 as non-related peptide antigen, HC as analyte).

Dissociation equilibrium constants (K_D_) were obtained by analyzing binding of peptide antigen SMAD2-P to cell-free produced IgG in steady state. IgG molecules as well as HC molecules as control were immobilized on Protein A coated sensor chips (Series S Sensor Chip Protein A, GE Healthcare) by injection of diluted SN2 (1:5 in PBST) for 40 min with flow rate of 2 µL/min. Subsequently, increasing concentrations of peptide antigen SMAD2-P (Stock in DMSO, peptide concentration range from 0.02–10 µM) were injected for 120 s followed by dissociation for 600 s (flow rate 20 µL/min). Additionally, a corresponding serial dilution of DMSO was applied on the chip under the same conditions. Referencing of binding curves was performed, using a control flow cell with antibody HC. The dissociation constant *K*_*D*_ was calculated from a plot of steady state binding levels against SMAD2P concentration. Data points were fitted by a steady state affinity fit curve.

## Electronic supplementary material

Supplementary Information
