# Supplementary material for: Cell-free synthesis of functional antibodies using a coupled in vitro transcription-translation system based on CHO cell lysates
Source: Sci Rep. 2017 Sep 20;7:12030. doi: 10.1038/s41598-017-12364-w (PMC5607253; doi:10.1038/s41598-017-12364-w)
Supplement: Supplementary file 1 — Supplementary Information [file 41598_2017_12364_MOESM1_ESM.pdf]

# Supplementary Information

## Cell-free synthesis of functional antibodies using a coupled *in vitro* transcription-translation system based on CHO cell lysates

M. Stech[1], O. Nikolaeva[1, 2], L. Thoring[1, 2], W.F.M. Stöcklein[1], D.A. Wüstenhagen[1], M. Hust[3], S. Dübel[3] and S. Kubick[1]

Affiliations
[1] Fraunhofer Institute for Cell Therapy and Immunology (IZI), Branch Bioanalytics and Bioprocesses (IZI-BB), Am Mühlenberg 13, 14476 Potsdam, Germany

[2] Technische Universität Berlin, Institut für Biotechnologie, Medizinische Biotechnologie, Gustav-Meyer-Allee 25, 13355 Berlin, Germany

[3] Technische Universität Braunschweig, Institute for Biochemistry, Biotechnology and Bioinformatics, Department of Biotechnology, Spielmannstr. 7, 38106 Braunschweig, Germany

To whom correspondence should be addressed: Dr. Stefan Kubick, Fraunhofer Institute for Cell Therapy and Immunology (IZI), Branch Bioanalytics and Bioprocesses (IZI-BB), Am Mühlenberg 13, 14476 Potsdam, stefan.kubick@izi-bb.fraunhofer.de, Tel: +49 331 58 187 -306 / Fax -119

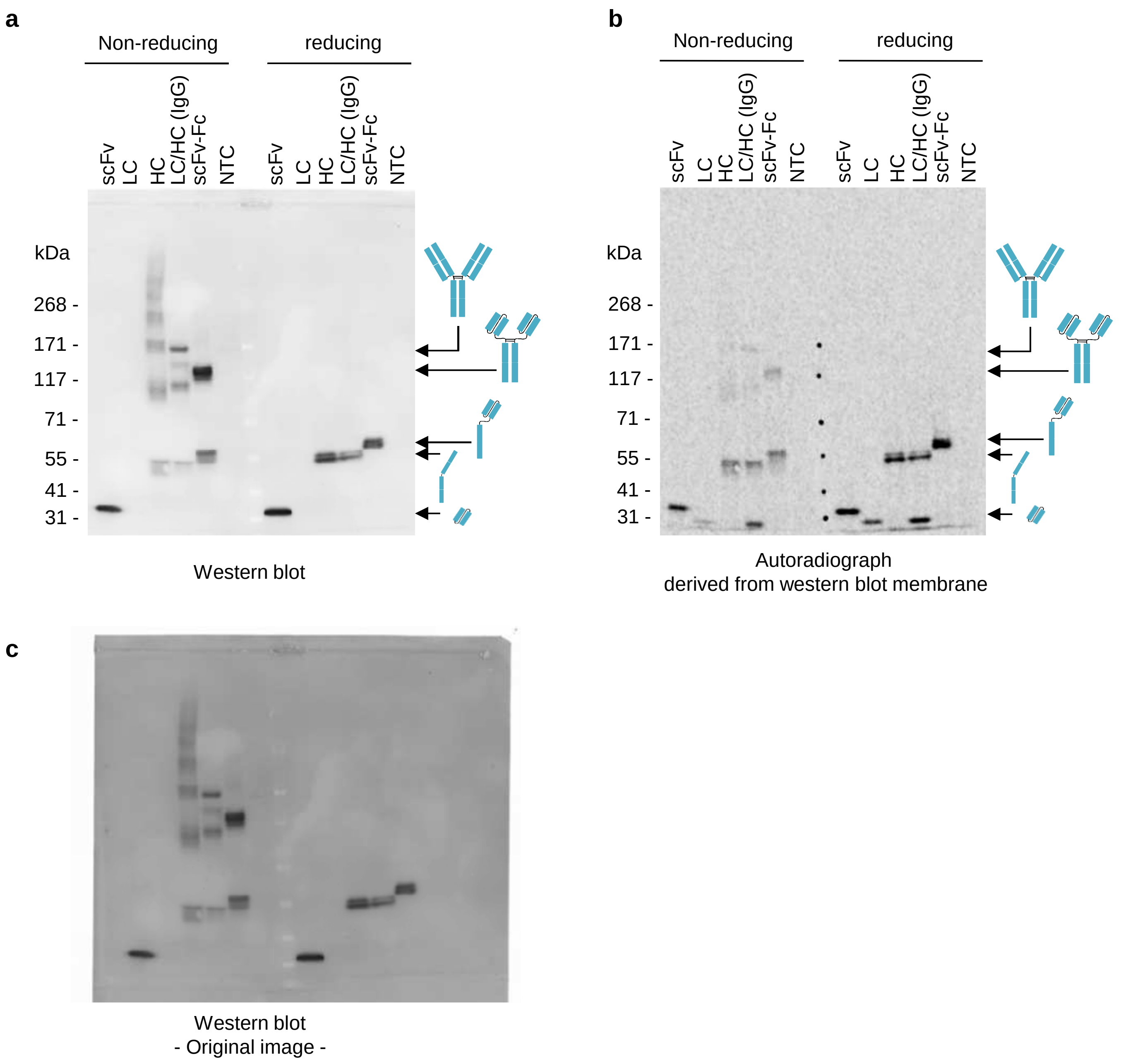


Supplementary Figure 1: Western blot of microsomal vesicles after cell-free synthesis of different antibody constructs. a) Detection was carried out by using "Mouse anti-c-Myc unconjugated monoclonal antibody" (Invitrogen) as primary and "Anti-mouse-IgG-HRP linked AB" as secondary antibody. Samples for SDS-PAGE were prepared by using either reducing sample buffer containing 50 mM DTT ("reducing") or non-reducing sample buffer ("non-reducing"). The western blot membrane has been adapted in contrast, brightness and sharpness for better visibility. The original image can be found in (c). b) Corresponding autoradiograph of the western blot membrane shown in (a).

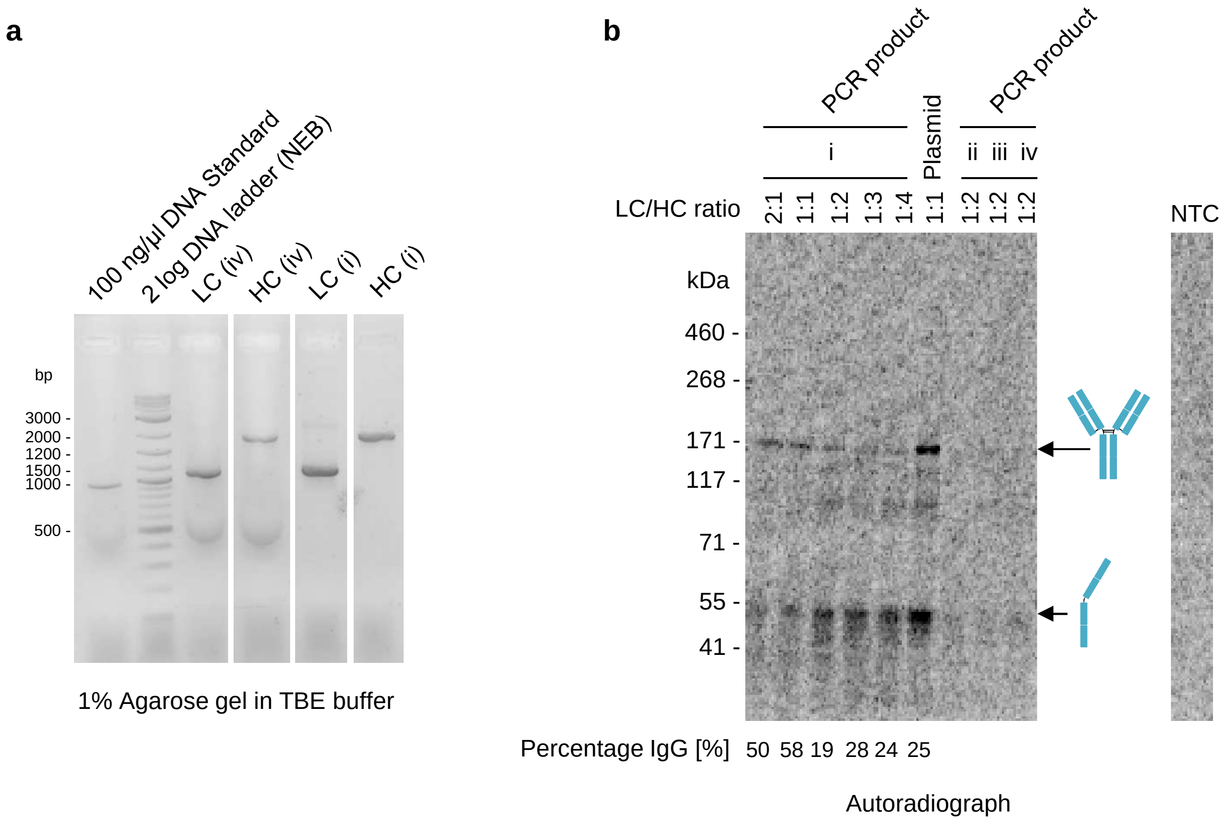


Supplementary Figure 2: Cell-free synthesis of antibodies based on PCR products as template. a) Agarose gel showing PCR amplified antibody light (LC) (1099 bp) and antibody heavy chain (HC) (1897 bp). In order to increase PCR product concentration, PCR reactions were purified by size exclusion chromatography (Invisorb® Fragment CleanUp) to desalt the sample and subsequently concentrated by using a centrifugal evaporator (Eppendorf Concentrator 5301). b) Autoradiograph derived from SDS PAGE gel showing the assembly of full length IgG (in lysate fraction MF) based on the use of PCR products as template. Cell-free reactions were supplemented with different ratios of concentrated and purified (i) LC and HC template: LC/HC ratios of 2:1 (25.4:12.7 nM),1:1 (19.1:19.1 nM), 1:2 (12.7:25.4 nM), 1:3 (9.5:28.6 nM) and 1:4 (7.6:30.5 nM). Usage of PCR products that have been concentrated, but not purified (ii), purified, but not concentrated (iii) or used directly without concentration and purification (iv) did not result in successful antibody synthesis, as only very faint protein bands were detectable in the autoradiograph. Exclusively the use of purified and concentrated PCR products (i) resulted in the detection of full length IgG. The following percentages of full length IgG were observed in dependency of the ratio of LC/HC template applied in the reaction: 50.28% (2:1), 58.24% (1:1), 18.52% (1:2), 28.45% (1:3) and 23.9% (1:4). NTC: No template control.

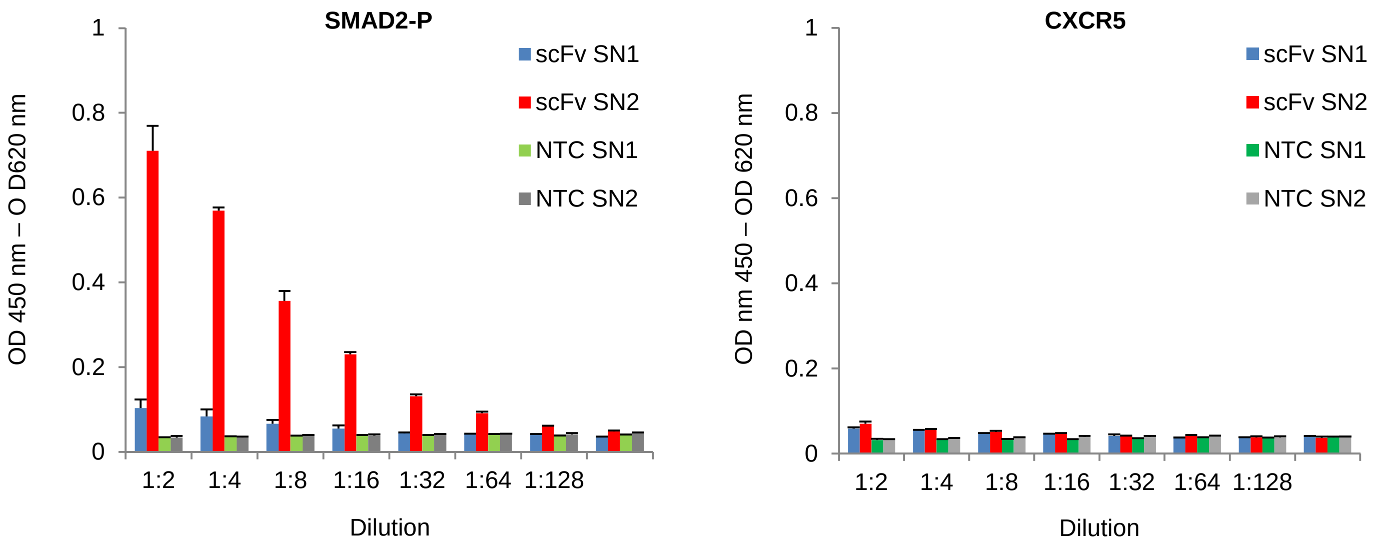


Supplementary Figure 3: ELISA analysis showing the specific binding of scFv „IIA4-His-c-Myc" to its antigen SMAD2-P, but not to CXCR5 (non-related antigen). scFvs present in the supernatant fraction after centrifugation (SN1) and the supernatant fraction (SN2) after detergent-based release of target proteins from the lumen of microsomal vesicles were tested in parallel. Starting concentrations (in dilution 1:2) of total cell-free synthesized protein were 0.0625 µg/mL. Standard deviations were calculated from duplicate analysis. NTC: No template control.

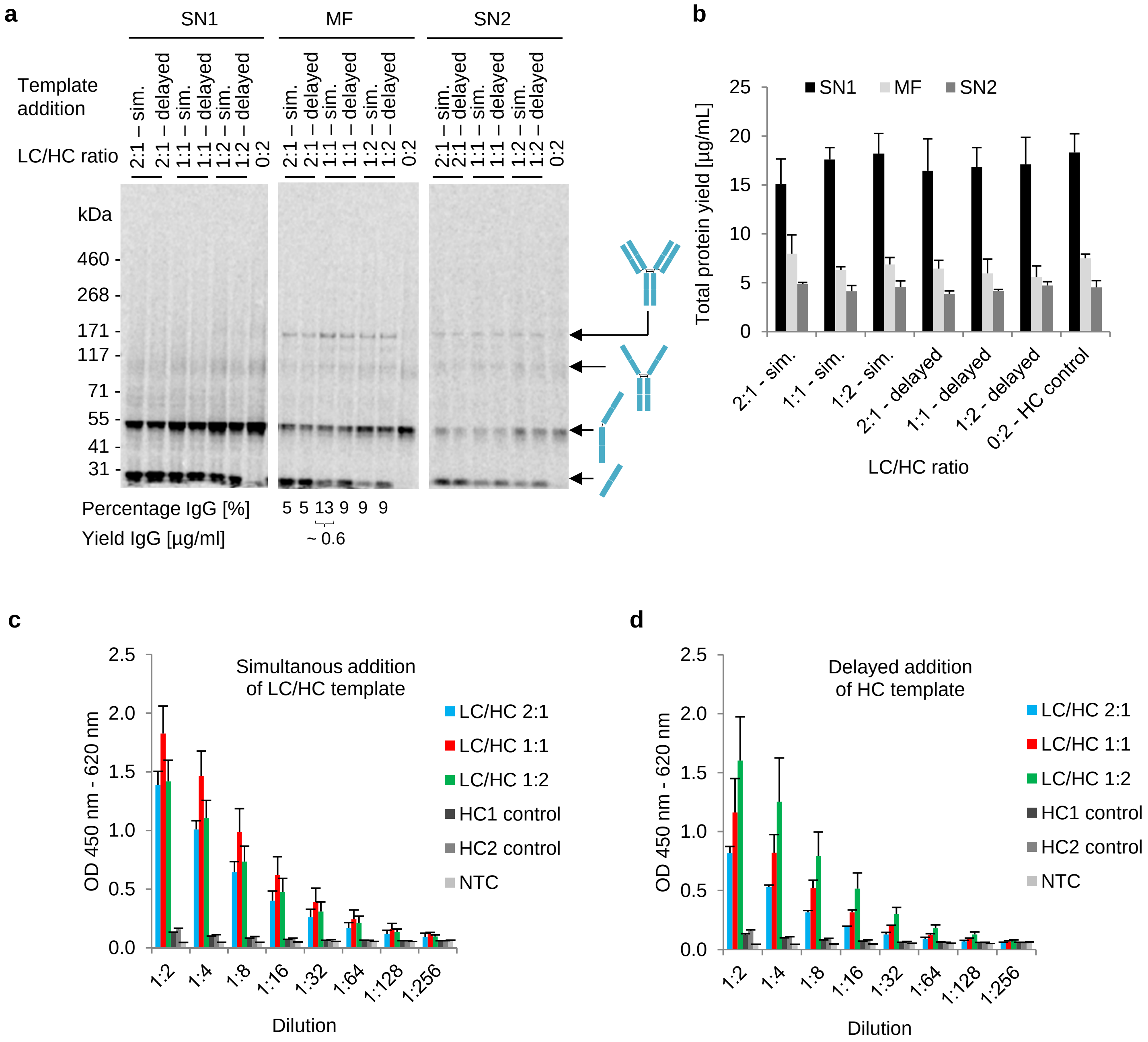


Supplementary Figure 4: Influence of template concentration and time-point of template addition on antibody chain assembly. Cell-free reactions were supplemented with different ratios of antibody light (LC) and antibody heavy chain (HC) plasmid template. LC/HC ratios of 1:1, 1:2, 2:1 correspond to plasmid ratios of 30 nM:30 nM, 30 nM:60 nM, 60 nM:30 nM. LC and HC templates were either added simultanously („sim.“) to start the cell-free reation or HC template was added with a 15 min delay („delayed“) compared to LC template addition. Antibody chain assembly was analyzed in the supernatant fraction after centrifugation (SN1), the microsomal fraction (MF) and the supernatant fraction (SN2) after detergent-based release of target proteins from the lumen of microsomal vesicles. a) Autoradiograph derived from SDS PAGE gel showing the assembly of antibody HC and LC to full lenght IgG molecules. b) Quantitative analysis of cell-free synthesized proteins in SN1, MF and SN2. c) and d) ELISA analysis showing the specific binding of LC/HC (IgG) to antigen SMAD2-P. Starting concentrations (in dilution 1:2) of LC/HC and HC control were between 0.23 – 0.3 µg/mL each. Standard deviations were calculated from duplicate analysis. NTC: No template control. HC1: 30 nM template DNA. HC2: 60 nM template DNA.

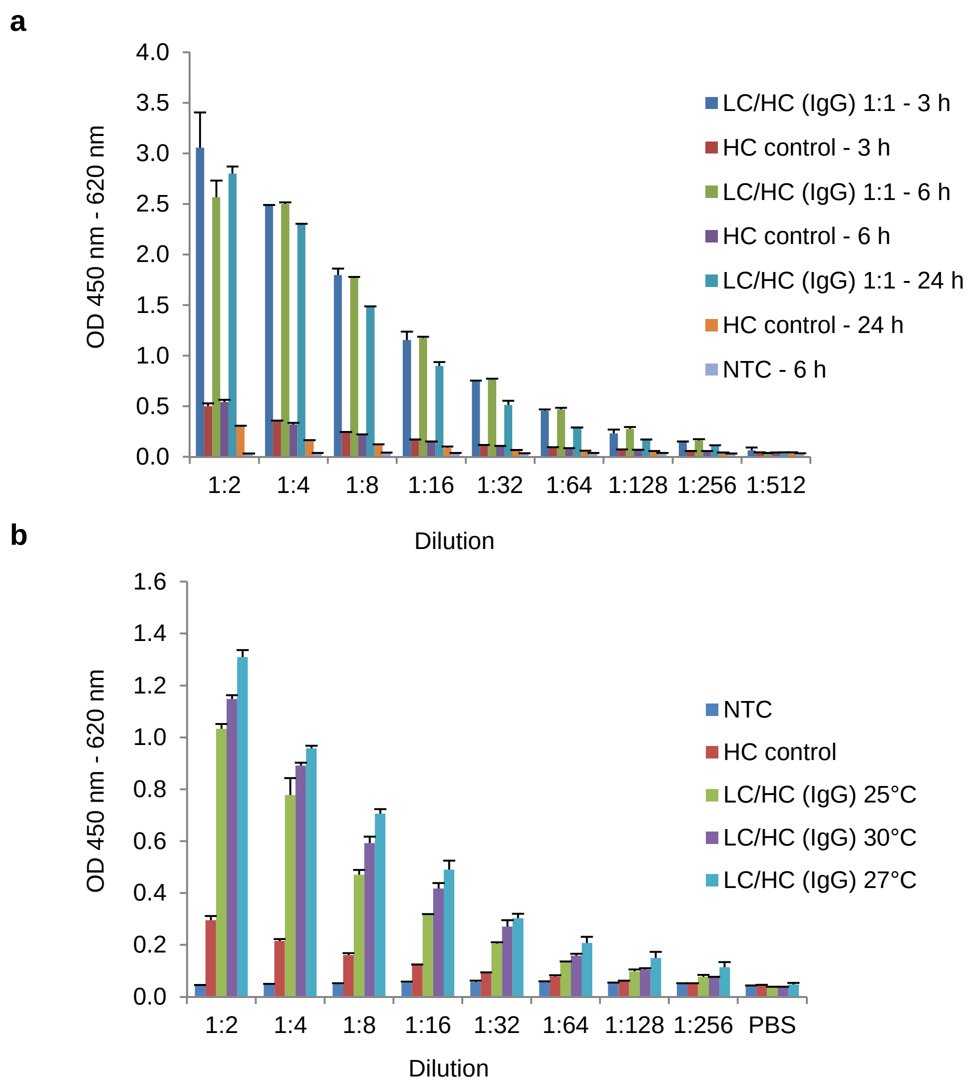


Supplementary Figure 5: Influence of incubation time and temperature on the activity of cell-free synthesized IgG. a) Batch-based protein synthesis reactions of LC/HC (IgG) and HC controls were incubated for 3 h, 6 h and 24 h and the effect on antibody functionality was analyzed by ELISA. Starting concentrations of total cell-free synthesized protein in all tested samples were 0.2 +/- 0.04 µg/mL (in dilution 1:2). b) Batch-based protein synthesis reactions of LC/HC (IgG) and HC controls were incubated at 25°C, 27°C and 30°C for 3 h and the effect on antibody functionality was analyzed by ELISA. Starting concentrations of cell-free synthesized protein in all tested samples were 0.25 +/- 0.04 µg/mL (in dilution 1:2). Standard deviations were calculated from duplicate analysis. LC: antibody light chain; HC: antibody heavy chain; NTC: No template control.

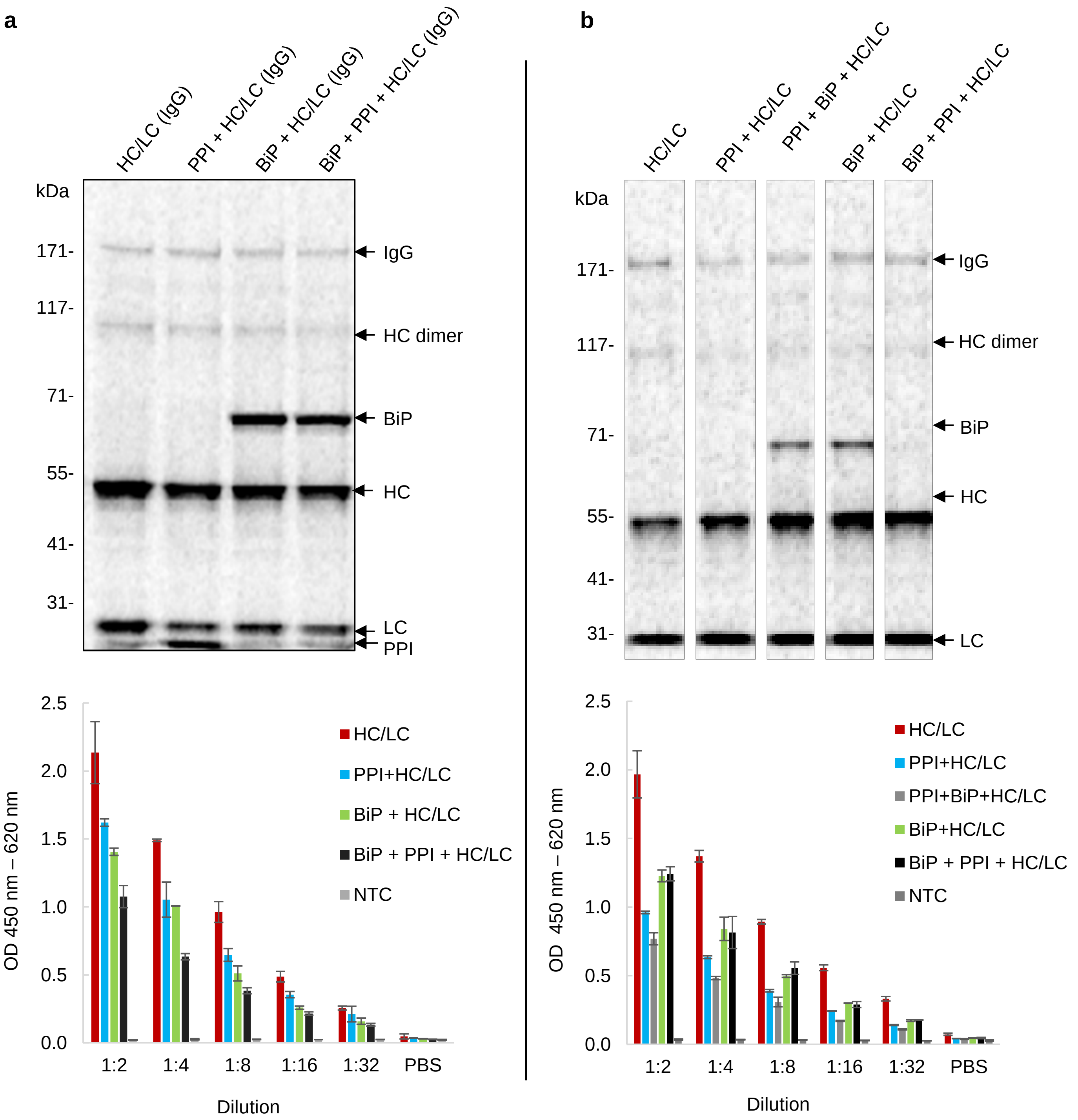


Supplementary Figure 6: Synthesis of LC/HC (IgG) in presence of cell-free synthesized Binding immunoglobulin protein (BiP) and Peptidyl-Prolyl-Isomerase (PPI). On the one hand, BiP, PPI, LC and HC were synthesized simultanously (coexpression) (a) or BiP, PPI, LC and HC were synthesized one after another (repetitive synthesis) (b). In the latter case microsomes containing cell-free synthesized BiP and/or PPI were harvested by centrifugation and used in a second translation reaction using fresh CHO lysate, in which the microsomes were depleted. Thus, *de novo* synthesis of HC/LC occured exclusively in microsomes primed with BiP and/or PPI. The figure legend indicates in which order and with which proteins the microsomes were primed. Analysis of the effect of BiP and PPI on HC/LC chain assembly was performed by SDS-PAGE and subsequent autoradiography. The corresponding ELISA analysis shows that HC/LC expression alone – without supplementation of BiP and PPI - results in the highest binding signal. Standard deviations were calculated from duplicate analysis. LC: Antibody light chain; HC: antibody heavy chain; NTC: No template control.

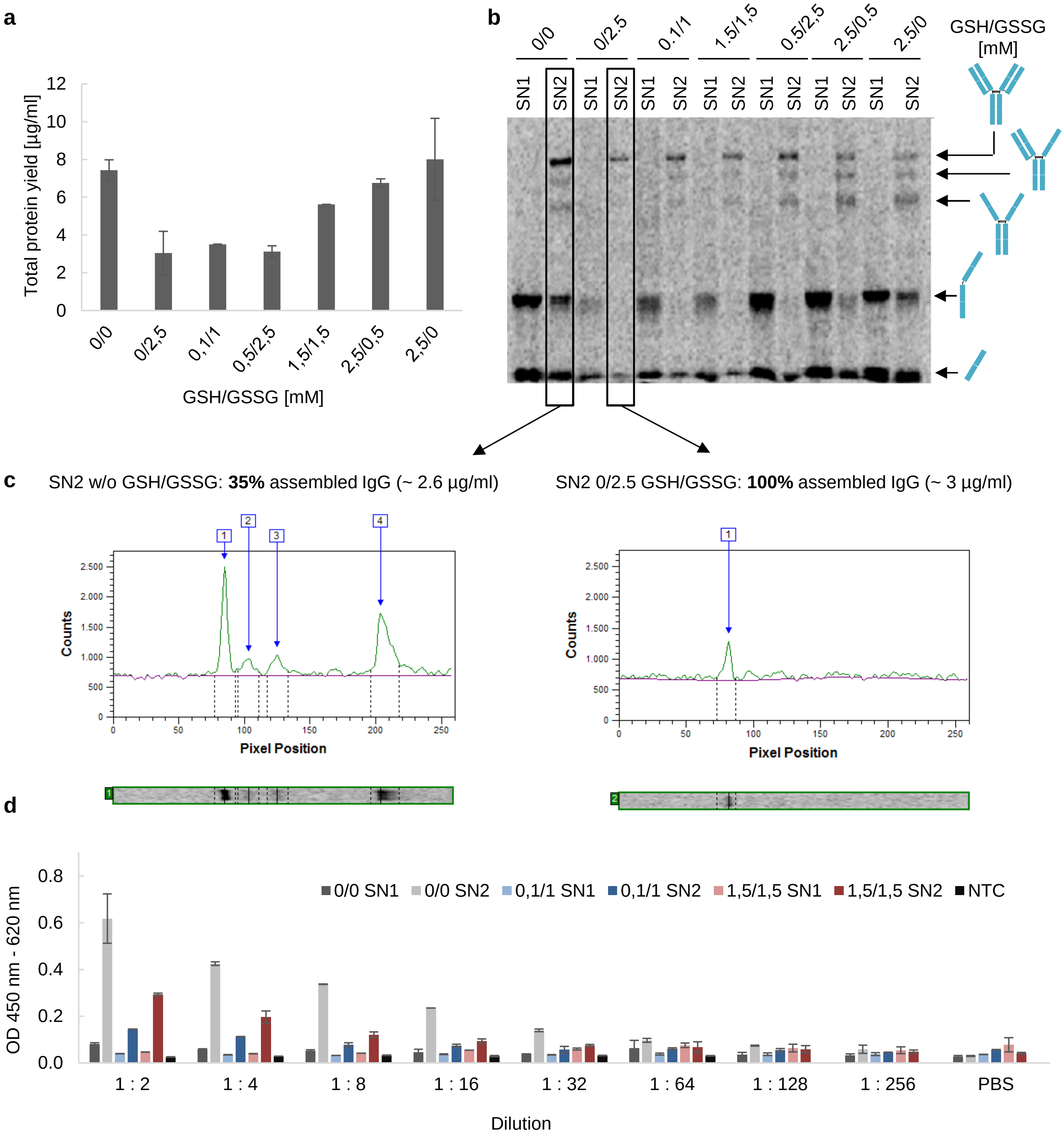


Supplementary Figure 7: Optimization of redox conditions in batch-based reactions by supplementing the *in vitro* translation reaction with reduced (GSH) and oxidized glutathione (GSSG). a) Influence of GSH/GSSG on the yields of *de novo* synthesized LC/HC (IgG) present in the microsomal fraction (MF). b) Autoradiograph derived from SDS-PAGE gel showing the influence of GSH/GSSG on the assembly of LC and HC to full length IgG analyzed in MF. c) Calculation of the percentage of intact IgG based on the observed band intensities in the autoradiograph shown in panel b. Densitometric evaluation was performed using ImageQuant TL software. d) Comparative ELISA analysis of antibodies present in the supernatant fraction after centrifugation (SN1) and the supernatant fraction (SN2) after detergent-based release of target proteins from the lumen of microsomal vesicles. Standard deviations were calculated from duplicate analysis.

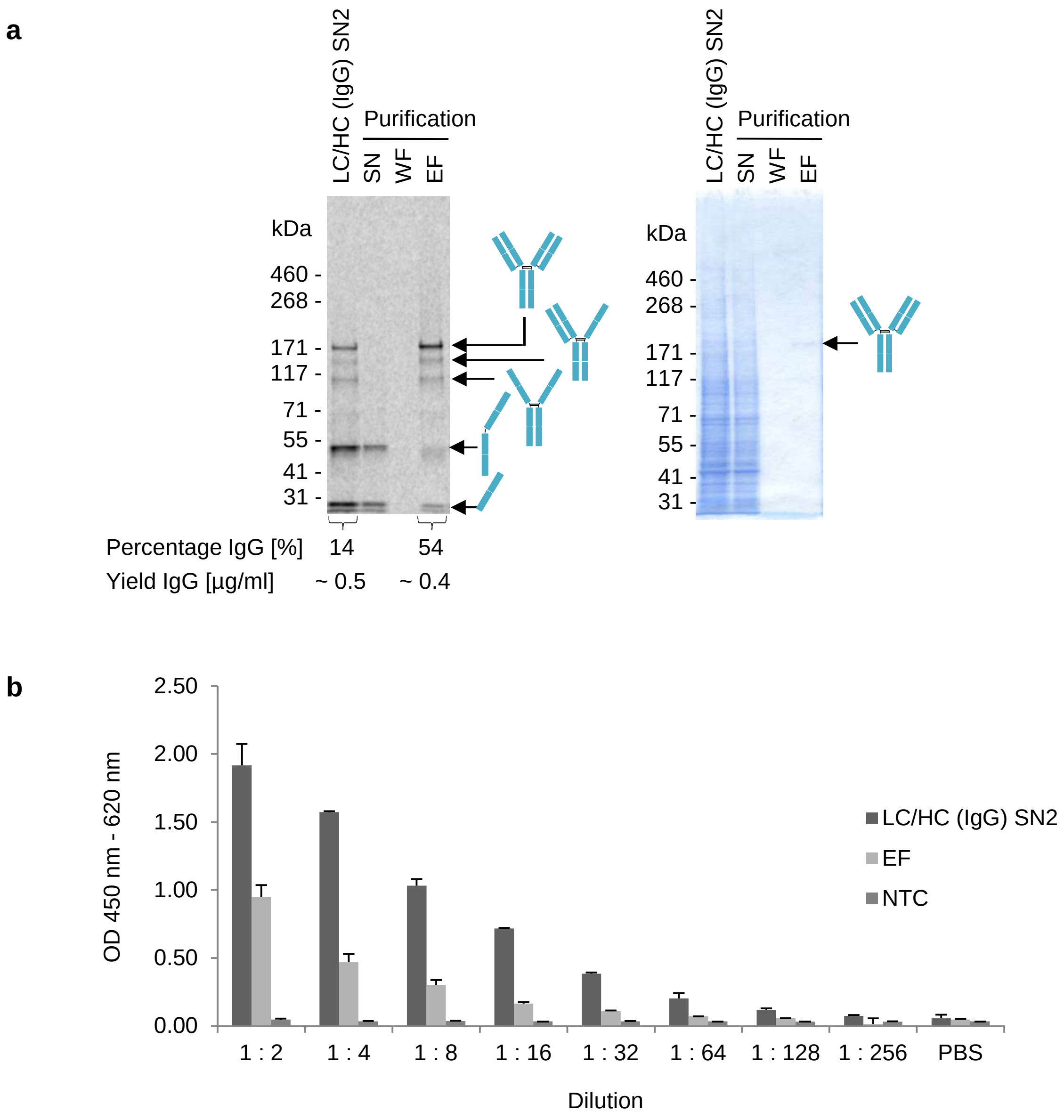


Supplementary Figure 8: Purification of cell-free synthesized IgG by using commercially available magnetic beads coupled to Protein G ("Immunoprecipitation Kit - Dynabeads® Protein G", Invitrogen). a) Autoradiograph (left) derived from Coomassie stained SDS-PAGE gel (right) to monitor purification of IgG using Protein G coupled magnetic beads (batch-based synthesis). The following fractions were analyzed: Supernatant fraction (SN2) after detergent-based release of target proteins from the lumen of microsomal vesicles, antibodies present in the supernatant after incubation with magnetic beads (supernatant), washing fraction (pool) and elution fraction (EF). Comparative ELISA analysis of antibodies present in SN2 and antibodies present in EF. Standard deviations were calculated from duplicate analysis.

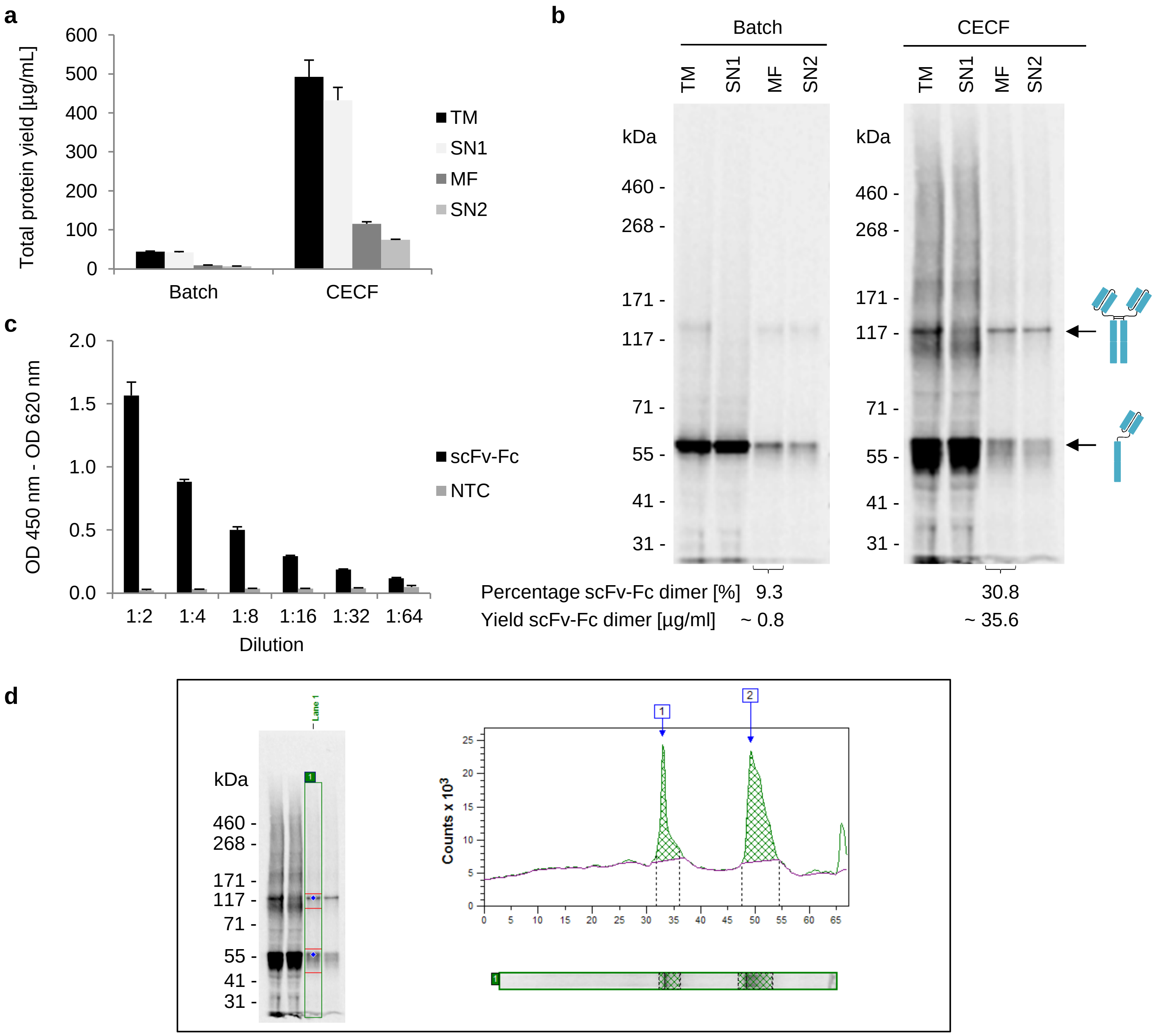


| Band No | Volume |
|---|---|
| 1 | 2895139.2 |
| 2 | 6508671.0 |

| Molecule | MW (kDa) | Number of leucines | Leucines/kDa | Band volume/(Leucines/kDa) | Band % |
|---|---|---|---|---|---|
| scFv-Fc dimer | 118.8 | 86.0 | 0.72 | 4000005.1 | **30.8** |
| scFv-Fc monomer | 59.4 | 43.0 | 0.72 | 8992561.5 | **69.2** |
| | | | | 12992566.6 | **100.0** |

| Protein yield in MF (µg/mL) | % | µg/mL | Molecule |
|---|---|---|---|
| 115.5 | **30.8** | 35.6 | scFv-Fc dimer |
| | **69.2** | 79.9 | scFv-Fc monomer |

Supplementary Figure 9: Comparison of batch- and CECF-based IRES-mediated cell-free synthesis of scFv-Fc. Protein synthesis reactions were performed in the presence of $^{14}$C-leucine for subsequent qualitative and quantitative analysis. a) Diagram showing protein yields determined in the complete translation mixture (TM), the supernatant fraction after centrifugation (SN1), microsomal fraction (MF) and supernatant fraction 2 after detergent solubilizazion (SN2). Standard deviations were calculated from triplicate analysis. b) Qualitative analysis of cell-free synthesized proteins by SDS-PAGE and subsequent autoradiography. c) ELISA analysis showing the specific binding of CECF-produced scFv-Fc to its antigen SMAD2-P. ELISA was performed with scFv-Fc present in SN2. Starting concentration in dilution 1:2 was approximatly 1.1 µg/mL total protein yield according to quantification via incorporation of $^{14}$C-leucine. Standard deviations were calculated from duplicate analysis. NTC: No template control. d) Calculation of protein yields of scFv-Fc monomers and dimers based on the observed band intensities in the autoradiograph and total protein yield determined in the microsomal fraction. Densitometric evaluation of scFv-Fc protein bands was performed using ImageQuantL software. Using the CECF mode 36 µg/mL fully assembled scFv-Fc could be determined corresponding to 31 % of total protein present in the microsomal fraction with 115.5 µg/mL set as 100%.

**a**

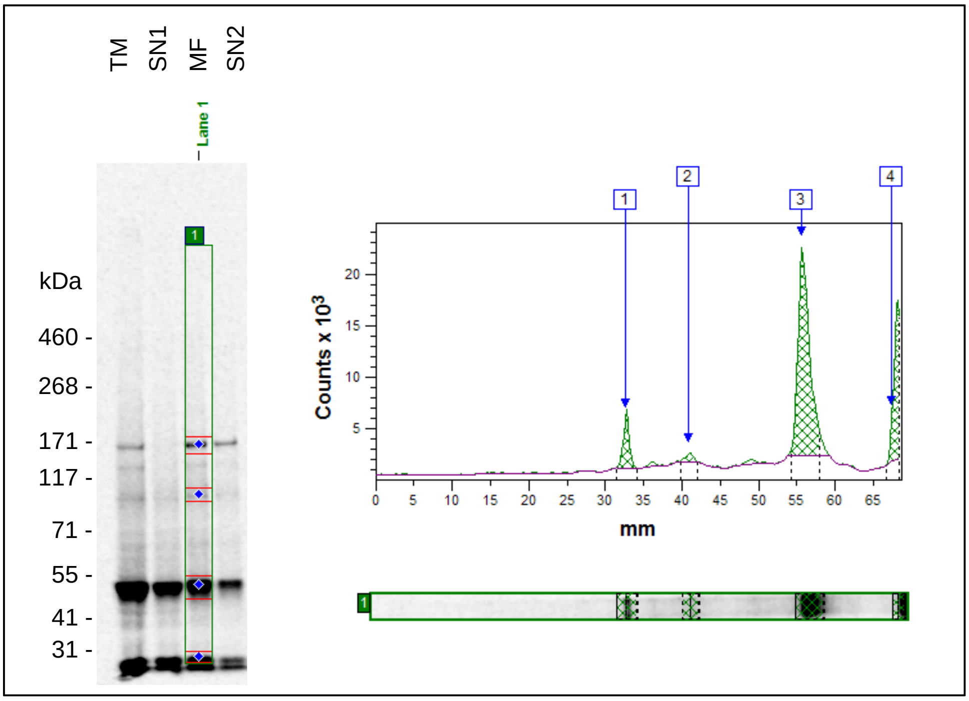


**b**

| Band No | Volume |
|---|---|
| 1 | 542817.0 |
| 2 | 104587.3 |
| 3 | 3849940.4 |
| 4 | 1248505.6 |

| Molecule | MW (kDa) | Number of leucines | Leucines/kDa | Band volume/(Leucines/kDa) | Band % |
|---|---|---|---|---|---|
| IgG | 160.9 | 118.0 | 0.7 | 739979.1 | **9.4** |
| HC dimer | 109.5 | 80.0 | 0.7 | 143101.6 | **1.8** |
| HC monomer | 54.7 | 40.0 | 0.7 | 5267680.9 | **67.2** |
| LC monomer | 25.6 | 19.0 | 0.7 | 1683051.2 | **21.5** |
| | | | | 7833812.8 | **100.0** |

| Protein yield in MF (µg/mL) | % | µg/mL | Molecule |
|---|---|---|---|
| 95.0 | 9.4 | 9.0 | IgG |
| | 1.8 | 1.7 | HC dimer |
| | 67.2 | 63.9 | HC monomer |
| | 21.5 | 20.4 | LC monomer |

Supplementary Figure 10: Calculating the yield of fully assembled IgG based on SDS-PAGE gels derived autoradiograms and TCA precipitation results presented in Figure 3b and 3c. a) Densitometric evaluation of protein bands detected in microsomal fraction (MF) using ImageQuantL software. b) Calculation of protein yields of antibody light chain (LC), heavy chain (HC), HC dimers and LC/HC hetero tetramers (IgG) based on the observed band intensities in the autoradiograph and total protein yield determined in the microsomal fraction (95 µg/mL). Using the CECF mode 9 µg/mL fully assembled IgG could be determined corresponding to 9.4 % of total protein present in the microsomal fraction with 95 µg/mL set as 100%.

**a**

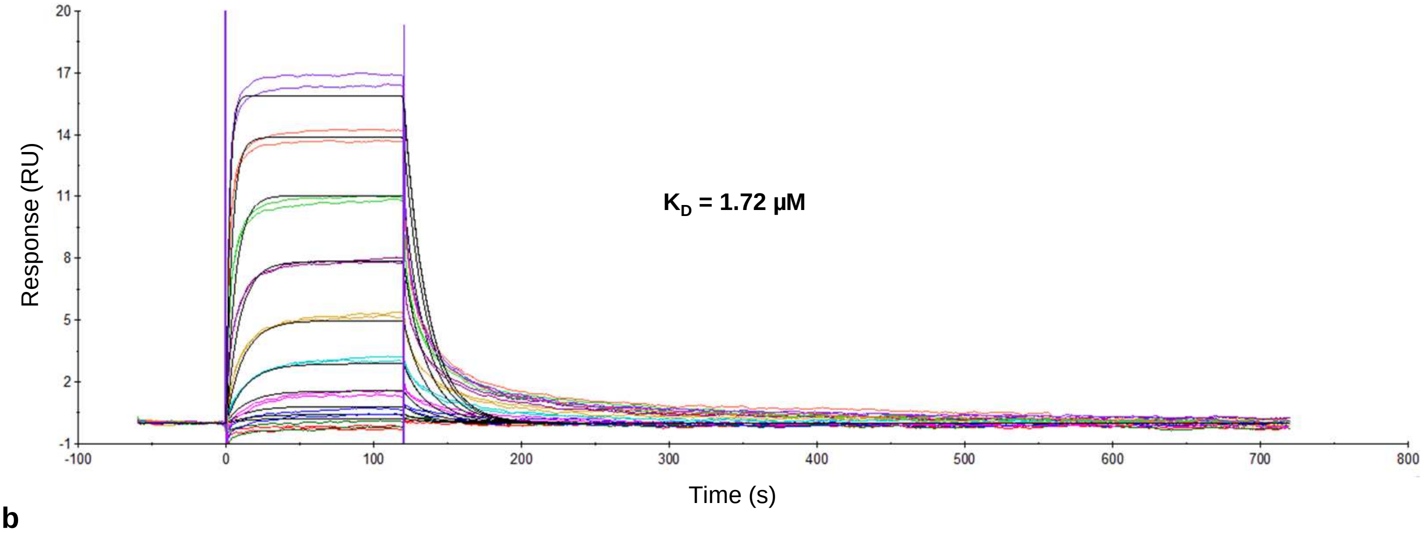


**b**

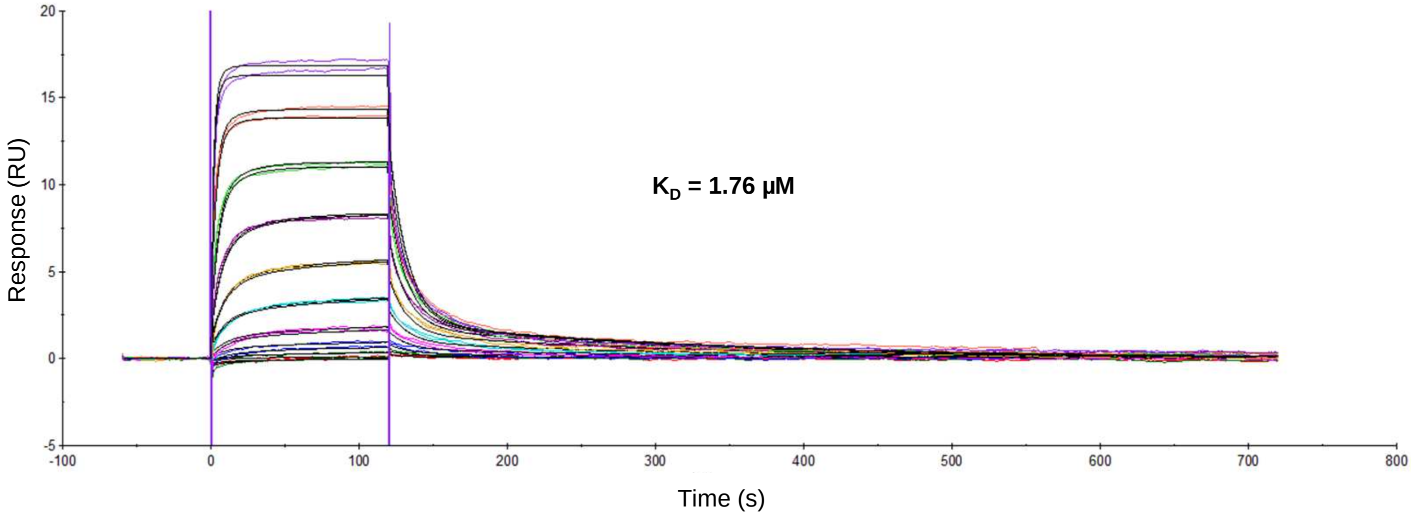


Supplementary Figure 11: Determination of dissociation equilibrium constants ($K_D$) by analyzing binding of peptide antigen SMAD2-P to cell-free synthesized IgG. a) Overlay of Biacore sensorgrams from two consecutive expriments showing the binding of peptide antigen SMAD2-P to immobilized IgG. Peptide antigen SMAD2-P was injected with increasing concentrations (10 µM, 5 µM, 2.5 µM, 1.25 µM, 625 nM, 313 nM, 156 nM, 78 nM, 39 nM, 20 nM) to determine the affinity. Plotting of Biacore sensorgrams using the kinetic fit model „1:1 binding" revealed a $K_D$ value of 1.72 µM ($R_{max}$: 18.6 RU, $chi^2$: 0.587). b) Plotting of Biacore sensorgrams using the fit model „heterogenous ligand" revealed a $K_D$ value of 1.76 µM ($R_{max}$: 12.3 RU, $chi^2$: 0.390; KD(2): 0.225 µM; $R_{max}$(2): 2.2 RU). Sensorgrams presented in a and b are reference subtracted. Referencing of binding curves was performed using a control flow cell with antibody heavy chain (HC). Since peptide dilutions were prepared from a stock solution in DMSO, corresponding serial dilutions of DMSO alone were applied on the chip under the same conditions. Effects of DMSO were shown to be neglectable (data not shown).

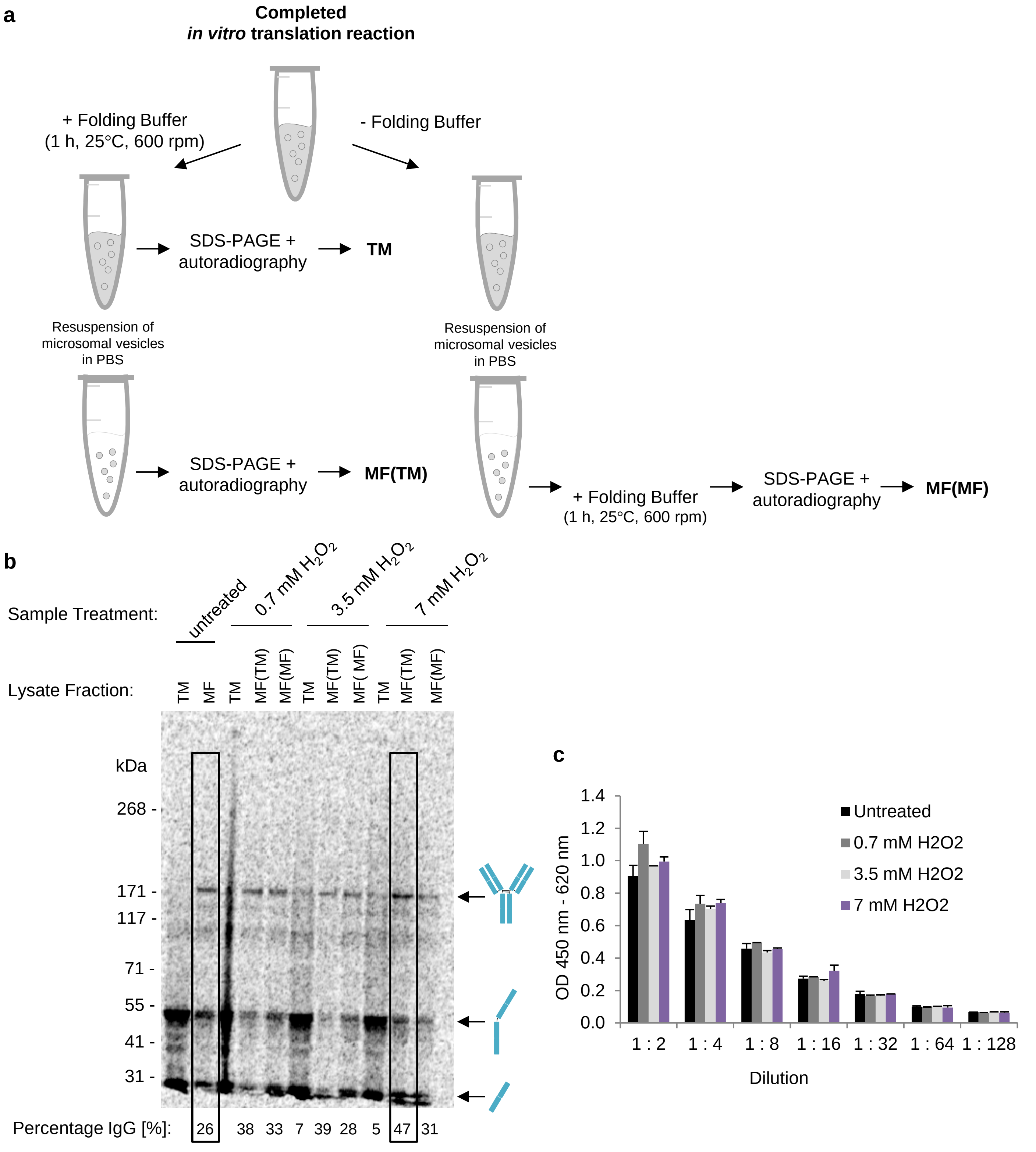


Supplementary Figure 12: Analyzing the effect of varying concentrations of $H_2O_2$ on the assembly of full length IgG. Cell-free synthesis of antibody heavy and light chain was performed using the batch-based CHO system. Cell-free reactions were incubated for 3 h (standard protocol). Aliquots of the complete translation mixture (TM) and the microsomal fraction (MF) were supplemented with 0.7 mM $H_2O_2$, 3.5 mM $H_2O_2$ and 7 mM $H_2O_2$ and incubated for 1 h at 25° and 600 rpm. Subsequently, samples were analyzed by SDS-PAGE and autoradiography. a) Schematic overview showing the sample preparation procedure. MF(TM): Lysate fraction MF was analyzed after treating TM with $H_2O_2$. MF(MF): Lysate fraction MF directly treated with $H_2O_2$. b) Autoradiograph derived from SDS-PAGE gel demonstrating that the supplementation of TM with 7 mM $H_2O_2$ (f.c.) resulted in an increase of fully assembled IgG from 26% (untreated) to 47%. c) ELISA analysis showing that cell-free synthesized antibodies treated with different concentrations of $H_2O_2$ are functionally active.
